# Scribble Modulates the MAPK/Fra1 Pathway to Disrupt Luminal and Ductal Integrity and Suppress Tumour Formation in the Mammary Gland

**DOI:** 10.1371/journal.pgen.1004323

**Published:** 2014-05-22

**Authors:** Nathan J. Godde, Julie M. Sheridan, Lorey K. Smith, Helen B. Pearson, Kara L. Britt, Ryan C. Galea, Laura L. Yates, Jane E. Visvader, Patrick O. Humbert

**Affiliations:** 1 Cell Cycle and Cancer Genetics Laboratory, Peter MacCallum Cancer Centre, East Melbourne, Victoria, Australia; 2 Sir Peter MacCallum Department of Oncology, University of Melbourne, Parkville, Victoria, Australia; 3 ACRF Stem Cells and Cancer Division, Walter and Eliza Hall Institute, Parkville, Victoria, Australia; 4 Department of Medical Biology, The University of Melbourne, Parkville, Victoria, Australia; 5 Department of Pathology, The University of Melbourne, Parkville, Victoria, Australia; 6 Metastasis Research Laboratory, Peter MacCallum Cancer Centre, East Melbourne, Victoria, Australia; 7 Department of Molecular Biology and Biochemistry, The University of Melbourne, Parkville, Victoria, Australia; National Cancer Institute, United States of America

## Abstract

Polarity coordinates cell movement, differentiation, proliferation and apoptosis to build and maintain complex epithelial tissues such as the mammary gland. Loss of polarity and the deregulation of these processes are critical events in malignant progression but precisely how and at which stage polarity loss impacts on mammary development and tumourigenesis is unclear. *Scrib* is a core polarity regulator and tumour suppressor gene however to date our understanding of *Scrib* function in the mammary gland has been limited to cell culture and transplantation studies of cell lines. Utilizing a conditional mouse model of *Scrib* loss we report for the first time that *Scrib* is essential for mammary duct morphogenesis, mammary progenitor cell fate and maintenance, and we demonstrate a critical and specific role for Scribble in the control of the early steps of breast cancer progression. In particular, *Scrib*-deficiency significantly induced Fra1 expression and basal progenitor clonogenicity, which resulted in fully penetrant ductal hyperplasia characterized by high cell turnover, MAPK hyperactivity, frank polarity loss with mixing of apical and basolateral membrane constituents and expansion of atypical luminal cells. We also show for the first time a role for Scribble in mammalian spindle orientation with the onset of mammary hyperplasia being associated with aberrant luminal cell spindle orientation and a failure to apoptose during the final stage of duct tubulogenesis. Restoring MAPK/Fra1 to baseline levels prevented *Scrib*-hyperplasia, whereas persistent *Scrib* deficiency induced alveolar hyperplasia and increased the incidence, onset and grade of mammary tumours. These findings, based on a definitive genetic mouse model provide fundamental insights into mammary duct maturation and homeostasis and reveal that *Scrib* loss activates a MAPK/Fra1 pathway that alters mammary progenitor activity to drive premalignancy and accelerate tumour progression.

## Author Summary

Polarity allows the specialization of cell function and is required to coordinate cell movements, differentiation, proliferation and apoptosis to build and maintain complex tissues such as the mammary gland. Disruption of polarity is a diagnostic criterion of cancer, but exactly how deregulation of core polarity genes contribute to cancer and at which stage polarity loss promotes breast cancer development *in vivo* is still poorly understood. To address this directly, we deleted the core polarity gene *Scrib* specifically in the mouse mammary gland. *Scrib* loss resulted in loss of tissue architecture and duct hyperplasia in mature but not pubescent mice. Onset of hyperplasia was associated with defective spindle orientations, a failure to apoptose and was sustained by high cell turnover and Ras/Erk/Fra1 MAPK pathway activation. *Scrib* deficiency activated progenitors and resulted in the excess growth of atypical luminal cells and the development of ductal and alveolar hyperplasia. Overall these mice exhibited an increased incidence, onset and grade of mammary tumours. These studies provide a definitive demonstration of the critical role played by core polarity genes in maintaining mammary epithelial integrity *in vivo*. This mouse model is a valuable tool for understanding the role of polarity in mammary development and the most initial stages of breast cancer.

## Introduction

The polarization of cells into distinct asymmetries is a central aspect of developmental cell biology. Through dictating the functional organization of cells within a tissue, polarity coordinates the movement, proliferation, differentiation and death of cells during tissue morphogenesis and homeostasis [1]–[3]. Polarity control is orchestrated by an intimate network of three mutually dependent complexes, the apically defined Par and Crumbs complexes and the basolateral Scribble complex [2]. In mammals, the Scribble complex consists of Discs large 1 through 4 (*Dlg1-4*), Lethal giant larvae 1 and 2 (*Lgl1/2*) and a single homologue of Scribble (*Scrib*) [4]. *Scrib* is a large (220-kDa) multidomain protein consisting of 16 leucine-rich repeat and 4 PDZ domains. As a critical component of the core polarity network, *Scrib* is required to establish distinct polarity configurations in response to spatiotemporal cues, but precisely how *Scrib* coordinates different cellular responses during a developmental program is less defined.

Scribble is likely to act as a signalling scaffold, interacting with various junctional/signalling components such as β catenin [5] and ZO-2 [6], the tumour suppressor APC [7], the ERK MAP kinase [8], polarity proteins VANGL2 and Lgl2 [9], and βPIX, a guanine nucleotide exchange factor for Rac [10]. *Scrib* modulation of cellular pathways during the development and homeostasis of epithelial organs is not well characterized and models to study how such core polarity genes coordinate key biological activities within a program of mammalian organ morphogenesis *in vivo* are required.

Postnatal development of the mouse mammary gland offers a unique system in which to study the role of polarity in epithelial organ morphogenesis. Mammary gland development and function requires several distinct polarity states [3]. For example, asymmetric cell divisions are required for stem/progenitor cells within the mammary epithelium to regulate cell diversification and to build balanced hierarchies of cells [3], [11]. Polarity control is also critical during duct morphogenesis where ductal elongation during puberty is driven by collective cell movements characterized by a transient depolarization of epithelial cells coupled to increased proliferation [12], [13]. The establishment of an apical-basal polarity state is then required for epithelial maturation and barrier function. Finally, migratory (front-rear) polarization occurs during wound healing and is also appropriated by breast cancer cells to allow invasion [3]. Despite being implicated in these various aspects of tissue development, our understanding of the role played by core polarity genes such as *Scrib* within these polarization processes *in vivo* remains limited. For example, control of symmetric and asymmetric cell divisions have been well characterized in *Drosophila* models and in other mammalian tissues [14], [15], however direct experimental evidence for asymmetric divisions and their regulation by core polarity genes during mammary gland development remains to be determined, despite several indirect approaches [16], [17].

Polarity control is also essential to maintain tissue integrity and homeostasis. Indeed, there has long been a strong correlation between loss of epithelial organization and malignant progression [18]. Scribble complex genes were originally identified as neoplastic tumour suppressors in Drosophila genetic screens, and studies using Drosophila genetics and 3D mammalian cell culture models suggest that disruption of core polarity regulators may contribute to tumourigenesis [4], [19]. In humans, deregulated expression and mislocalisation of Scribble complex members are associated with several epithelial cancers [20]–[25], including breast cancer [26]–[29], and human SCRIB is targeted by oncogenic viruses for degradation [30]. To date, our understanding of the role of *Scrib* in mammary tumourigenesis has been limited to cell culture and transplantation models [29], [31]. Whilst transplantation and *in vitro* studies have rapidly determined a potential role for Scribble in mammary tumourigenesis, transplantation models fail to mimic pre-malignancy or spontaneous tumour development. Our laboratory has therefore established a unique conditional model for Scribble loss. We have recently used this conditional model to show a requirement for *Scrib* in the prostate and lung where targeted *Scrib*-loss in the prostate led to prostate intraepithelial neoplasia and in both lung and prostate setting, cooperated with the K-Ras oncogene in tumour progression [25], [32].

In the present study, we have conditionally depleted *Scrib* in the mammary gland to demonstrate how Scribble plays a tumour suppressive role during mammary gland development and homeostasis. These studies highlight specific roles for *Scrib* including regulation of spindle orientation, Ras/MAPK signalling and control of basal to luminal transition and establish polarity suppression of Fra1 as an important barrier to the initiation of premalignant lesions in the breast. This mouse model is a valuable tool for understanding the role of cellular polarity in mammary development and breast cancer progression.

## Results

### Conditional deletion of *Scrib* in the mammary gland alters branching morphogenesis and causes duct hyperplasia

**Figure 1:**
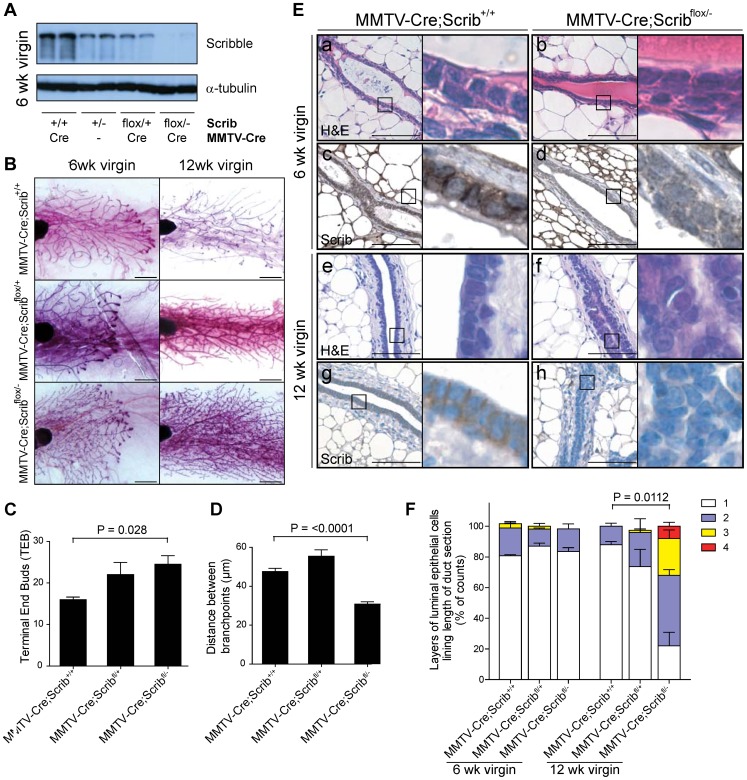
*Scrib* loss in the mammary gland results in duct hyperplasia. **A.** Confirmation of *Scrib* deletion in mammary glands of *MMTV-Cre;Scrib^flox/−^* 6 week old virgin mice by immunoblotting. **B.** Mammary whole mount analysis of 6 and 12 week old *MMTV-Cre*, *MMTV-Cre;Scrib^flox/+^* and *MMTV-Cre;Scrib^flox/−^* virgin mice. Scale bar = 2 mm. **C.** Quantitation of increased terminal end bud number during duct morphogenesis of peripubescent *MMTV-Cre;Scrib^flox/−^* 6 wk virgin mice compared to control mice. ± SEM. Mann Whitney t-test, (n = 5–9). **D.** Quantitation of increased branching in mammary glands of post-pubescent *MMTV-Cre;Scrib^flox/−^* 12 wk virgin mice compared to control mice. ± SEM. Mann Whitney t-test, (n = 3–6). **E.** Histological analysis by H&E staining show normal duct formation in peri-pubescent 6 wk virgin *MMTV-Cre* (a) and *MMTV-Cre;Scrib^flox/−^* (b) mice. Longitudinal sections of mature mammary ducts show organized epithelial bi-layer in 12 wk virgin *MMTV-Cre* control mice (e), whereas mammary ducts from 12 wk *MMTV-Cre;Scrib^flox/−^* mice (f) have a highly disorganized epithelium with luminal occlusion resulting from an abundance of poorly differentiated and multilayered mammary epithelial cells. IHC shows basolateral localization of Scribble in luminal epithelial cells of 6 (c) and 12 week old (g) *MMTV-Cre* control mice and validates *Scrib* loss in mammary epithelium of 6 (d) and 12 week old (h) *MMTV-Cre;Scrib^flox/−^* mice. Scale bar = 100 µm. **F.** Quantitation of multilayering phenotype performed by counting layers of luminal epithelial cells along length of longitudinal duct sections. ± SEM. Mann Whitney t-test, (n = 3–5). See also Figure S1.

To specifically delete *Scrib* in the mammary gland we bred mice carrying *Scrib^flox/+^* conditional and germline *Scrib^+/−^* deletion alleles [25] with *MMTV-Cre* transgenic mice [33]. *MMTV-Cre* directs efficient excision of floxed genes in both luminal (>80%) and basal/myoepithelial (99%) cell types in the mammary gland from day 6 post-partum [34], [35]. Loss of Scribble protein in the mammary glands of *MMTV-Cre;Scrib^flox/−^* mice was confirmed by immunoblotting and immunohistochemistry (Figures 1A, 1E (d), (h)). Partial loss was observed in mammary glands of mice with genotypes heterozygous for *Scrib* (*MMTV-Cre;Scrib^flox/+^* and *No Cre;Scrib* ^+/−^) (Figure 1A). Consistent with previous observations in other epithelial tissues, Scribble basolateral localization was detected by IHC in the mammary epithelium of wildtype and *MMTV-Cre* control mice (Figures 1E (c), (g)) [25], [31].

To investigate the developmental impact of *Scrib* loss on branching morphogenesis we examined whole-mount preparations of inguinal mammary glands from pubescent (6 week) and mature (12 week) *MMTV-Cre*, *MMTV-Cre;Scrib^flox/+^* and *MMTV-Cre;Scrib^flox/−^* virgin mice. During puberty, the rudimentary mammary gland branches and elongates throughout the mammary fat pad via highly proliferative terminal end bud (TEB) structures consisting of mammary progenitor populations. At 6 weeks, ductal trees of *MMTV-Cre;Scrib^flox/−^* virgin mice showed significantly higher numbers of TEB's (Figure 1B, quantitated in 1C). Following maturation at 12 weeks of age, TEBs were absent and the ductal network had extended throughout the fat pad of all mice examined. Excessive branching however was evident in *Scrib*-deficient mammary glands (Figure 1D), as indicated by the distances between branch points (excluding small side branches). Whilst histological analysis revealed that loss of Scribble in the developing mammary gland did not immediately impact on duct formation in 6 week virgin mice (Figure 1E (a–d)), gross morphological defects were observed in mammary ducts of *MMTV-Cre;Scrib^flox/−^* virgin mice by 12 weeks. *Scrib*-deficient hyperplastic lesions displayed a loss of ductal structure, luminal space and concomitant diffuse intraductal hyperplasia which consisted of disorganized epithelial cell layers of at least 3–4 cells with nuclear and cellular pleomorphism (Figures 1E (e–h)). Multilayering was observed in the majority of mammary duct cross sections of *MMTV-Cre;Scrib^flox/−^* mice and was significantly increased compared to *MMTV-Cre* control mice (Figure 1E and quantitated in 1F). We did not detect any phenotypes associated with *Scrib* loss in other tissues and ductal multilayering was not observed in *MMTV-Cre;Scrib^flox/+^* or *MMTV-Cre;Scrib^+/−^* heterozygous mice (Figure 1F, Figure S1A). Taken together, our data demonstrate that *Scrib* is essential for late stage ductal morphogenesis, maturation and homeostasis.

### Scribble deficient lesions display loss of polarity and expansion of atypical intraluminal mammary epithelial cells

To identify the key cellular functions mediated by Scribble that are required for proper mammary duct formation, we conducted an extensive analysis of the differentiation, organization, survival and proliferation of developing *Scrib*-deficient mammary glands.

**Figure 2:**
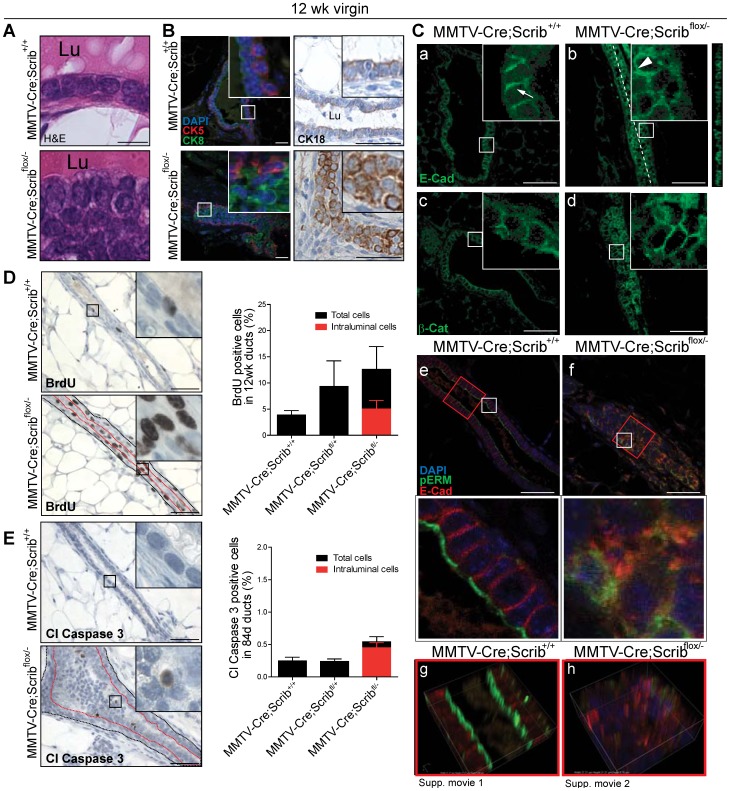
*Scrib*-deficient lesions are characterized by polarity loss and the expansion of rapidly cycling poorly differentiated luminal mammary epithelial cells. **A.** Histopathology by H&E staining of well differentiated luminal epithelial cells from *MMTV-Cre* control mice which are cuboidal with small tightly compacted nuclei and consistent cell-cell adhesion boundaries. Mammary ductal cells of *MMTV-Cre;Scrib^flox/−^* virgin mice are no longer cuboidal, have loosely packed atypical nuclei, increased cytoplasm and inconsistent cell-cell adhesions (Lu: Lumen) **B.** Immunostaining in mammary ducts of 12 week mice show normal distribution of luminal (Cytokeratin 8, green and Cytokeratin 18 by IHC), and basal (Cytokeratin 5, red) cell populations in ducts from *MMTV-Cre* mice, whereas an expansion of Cytokeratin 8/18 luminal cells is observed in ducts from *MMTV-Cre;Scrib^flox/−^* mice. Scale bar = 50 µm. **C.** Immunofluorescence of adhesion junctional proteins E-Cadherin (a,b) and β-catenin (c,d) in 12 wk virgin mice show normal lateral staining in luminal epithelial cells of *MMTV-Cre* mice (arrow) and ectopic randomized membrane staining in mammary epithelial cells of *MMTV-Cre;Scrib^flox/−^* mice (arrowhead). Z-projection included for E-cadherin staining. Loss of membrane segregation in mammary ducts of 12 week *MMTV-Cre;Scrib^flox/−^* virgin mice by confocal immunofluorescence microscopy of apical membrane marker pERM (green) and E-cadherin (red) (e, f). Red squares indicate regions in which 3D reconstructions were made from confocal z-series (g, h,)(Movie S1, S2) Scale bar = 50 µm. **D.** IHC and quantitation of proliferation (BrdU) and **E.** apoptosis (Cleaved Caspase 3) in ducts of 12 week virgin mice. (Additional quantitation of intraluminal cells not in contact with myoepithelial/basement membrane region was performed in *MMTV-Cre;Scrib^flox/−^* mice and expressed as percentage of total luminal epithelial cell population. ± SEM. Mann Whitney t-test, (n = 3–5). Scale bar 100 µm. See also Figure S1.

Fully differentiated luminal epithelial cells that line mammary ducts exhibit apical-basal polarization, and are cuboidal with centralized nuclei, minimal cytoplasm and lateral boundaries in full contact with neighbouring cells [36]. Cells accumulating within the ducts of *MMTV-Cre;Scrib^flox/−^* mutant mice lacked many of these aspects of epithelial differentiation: cells were poorly differentiated, with a prevalence of loosely packed rounded cells with larger atypical nuclei and increased cytoplasm (Figure 2A). To examine the cell composition of *Scrib*-deficient mammary ducts, we immunostained for the luminal lineage markers Cytokeratin 8 and 18 (CK8, green; CK18 IHC) and basal/myoepithelial marker Cytokeratin 5 (CK5, red) (Figure 2B). In contrast to the usual bilayered ductal epithelium composed of a luminal cell layer surrounded by a basal myoepithelial layer (*MMTV-Cre* control mice, Figure 2B), we observed a dramatic expansion of CK8/18 positive luminal cells in *Scrib*-deficient mammary ducts (Figure 2B). This abnormal increase in CK8/18 positive cells accounts for the bulk of the poorly differentiated intraluminal cells in *Scrib*-deficient mammary ducts. Similar results were obtained by CK8/CK14 dual staining (Figure S1B).

Mammary epithelial differentiation is characterized by the establishment of apical basal polarity, the formation of mature tight junctions (TJs) and adherens junctions (AJs), and the specification of distinct apical and basolateral membrane identities [3]. *Scrib* has been implicated in TJ formation [6], [37], [38]. Furthermore, Scribble is known to interact with or regulate the function of key junctional complex components such as E-cadherin and β-catenin [5], [6], [39]. Therefore we determined the impact of *Scrib* loss on the junctional integrity of mammary epithelial cells and found the apical membrane and junctional localization of ZO-1 was maintained in the absence of *Scrib* (Figure S1C). Ultrastructural analysis of cell-cell junctions on mammary ducts of *Scrib* mutant mice confirmed that TJs and desmosomes are still able to form. However a marked decrease in the electron density of TJs and desmosomes in ducts of *MMTV-Cre;Scrib^flox/−^* mice was observed suggesting minor defects in junction formation (Figure S1D & Procedures S1). Whilst *Scrib*-deficient mammary epithelial cells were multilayered and varied in cell shape similar to mammary epithelial cells undergoing morphogenesis, electron microscopy did not reveal interdigitating membrane extensions that are characteristic of normal multilayered morphologically active epithelial cells of the terminal end bud [13]. When adherens junctions were examined, in contrast to the lateral enrichment of junctional proteins observed in control mice (Figures 2C (a), (c)). white arrow), hyperplastic ducts of *MMTV-Cre;Scrib^flox/−^* mutant glands revealed a random distribution of E-cadherin and β-catenin across the membranes of epithelial cells with irregular enrichment at regions of cell-cell contact (Figures 2C (b), (d) white arrowhead). We did not observe increased nuclear β-catenin staining, suggesting that the Wnt pathway is not activated in *Scrib*-deficient tissue. Consistent with previous RNAi studies of Scribble in cell lines [39], [40], western blot analysis determined that *Scrib* loss did not significantly impact on the protein levels of E-cadherin or β-catenin (Figure S1E). To further examine apical basal polarity, we evaluated the separation of distinct membrane identities in *Scrib*-deficient epithelial cells by dual immunofluorescence for E-cadherin and for the apical membrane domain marker p-ezrin(Thr567)/p-radixin(Thr564)/p-moesin(Thr558) (pERM) in ducts of 12 week virgin mice. All *Scrib*-deficient ducts exhibited highly disorganized aggregated pools of membrane domains where apical and lateral markers were co-localized, suggesting *Scrib*-deficient mammary cells are unable to respond appropriately to contextual cues and directional cellular processes to establish apical-basal polarity (Figures 2C (e–h), see Movies S1 and S2). Similar staining was also observed using the apical membrane marker and proto-oncogene MUC-1 (Figure S1F). Together these data indicate that loss of Scribble in the mammary gland leads to severe disruptions in apical-basal polarity associated with altered ductal architecture.

We next evaluated proliferation and apoptosis in the mature ducts of 12 week old *MMTV-Cre;Scrib^flox/−^* and *MMTV-Cre;Scrib^flox+^* mutant mice using BrdU and Cleaved Caspase 3 respectively. We detected higher rates of proliferation in *Scrib*-deficient ducts compared to ducts from *MMTV-Cre* control mice (Figure 2D), supporting previous work in *Scrib*-deficient prostate lesions [25]. The development of ductal hyperplasia was also accompanied by increased apoptosis which occurred entirely within the mislocalised intraluminal cell population (Figure 2E). However, we observed no evidence of increased autophagy as measured by lack of autophagosomes by electron microscopy or increase in LC3-II conversion (Figure S1D; Data not shown). Therefore although *Scrib*-deficient luminal cells initially survive following delamination from contact with the myoepithelial/basement membrane layer, they are unable to persist in the intraluminal space as would be observed in more advanced premalignant lesions such as ductal carcinoma *in situ* (DCIS). *Scrib*-deficiency induced hyperplasia therefore recapitulates the most initial stages of pre-malignancy in the breast.

### *Scrib* coordinates early tissue organization and cell division orientation during duct maturation and homeostasis

**Figure 3:**
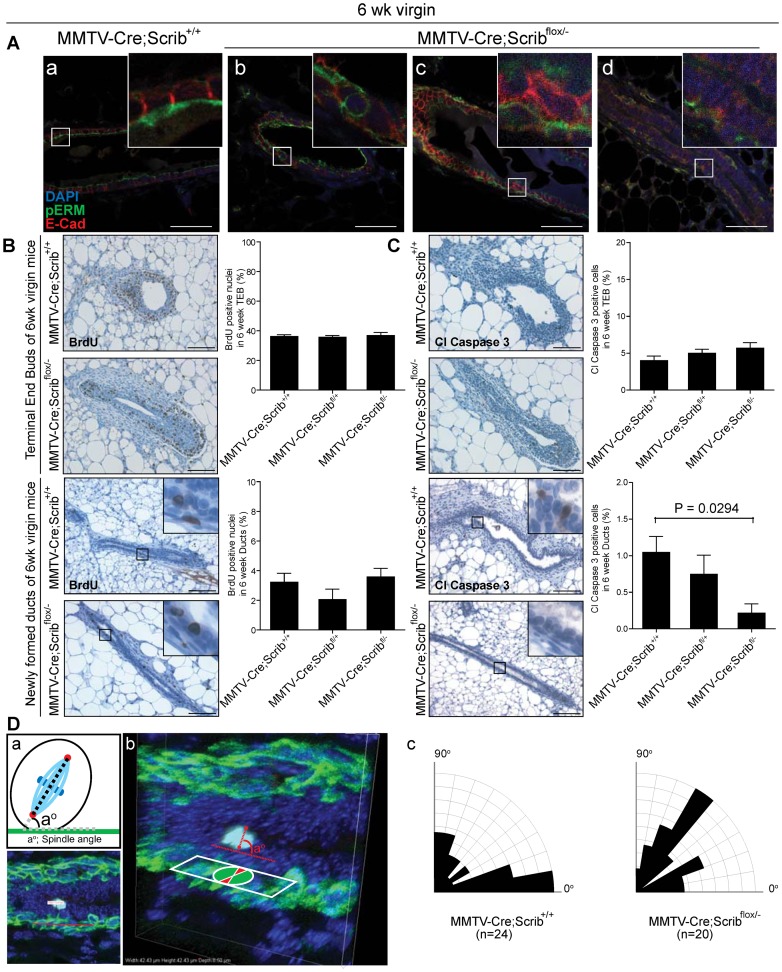
Hyperplasia is preceded by defects in cell polarity, apoptosis and spindle orientation during the remodelling and maturation of *Scrib*-deficient mammary ducts. **A.** Confocal immunofluorescence microscopy of apical membrane marker pERM (green) and lateral membrane marker E-cadherin (red) show polarised membrane segregation in mammary ducts of 6 week *MMTV-Cre* control mice (a), and partial to complete disruption of polarity in ducts of *MMTV-Cre;Scrib^flox/−^* mice (b, c, d). Scale bar = 50 µm. **B.** IHC and quantitation of proliferation (BrDU) and **C.** apoptosis (Cleaved Caspase 3) in terminal end buds and immature ducts of 6 week old *MMTV-Cre*, *MMTV-Cre;Scrib^flox/+^* and *MMTV-Cre;Scrib^flox/−^* virgin mice. ± SEM. Mann Whitney t-test, (n = 3–5, 5–9 TEB/mouse). Scale bar 100 µm. **D.** Spindle orientation of dividing luminal cells in 6 wk virgin mice. Mitotic cells were identified by PHH3 (light blue) immunofluorescence and spindle angle determined by centrosome positioning (pericentrin, red) compared to the lower layer of myoepithelial cells (CK5, green) (a). Representative images of a division within a *Z*-stack (b). Rosette plots showing percentage of spindle orientations in ducts of *MMTV-Cre* and *MMTV-Cre;Scrib^flox/−^* mice (c).

To address how *Scrib* depletion leads to hyperplasia and loss of tissue architecture, we first examined cell polarity in *Scrib*-deficient mammary ducts prior to the onset of multilayering in 6 week old virgin mice. As expected two distinct lateral (E-cadherin, red) and apical (pERM, green) membrane regions were present in ducts of *MMTV-Cre* control mice (Figure 3A (a)). However ducts of *MMTV-Cre;Scrib^flox/−^* mutant mice displayed varying degrees of polarity loss in the absence of multilayering, with some individual cells exhibiting expanded apical membrane domain regions (Figure 3A (b)), and others lacking lateral enrichment of E-cadherin (Figure 3A (c)). In some instances, completely disorganized aggregates of membrane domains randomly distributed across cell surfaces were observed in epithelial cells lining the ducts of 6 week virgin *MMTV-Cre;Scrib^flox/−^* mutant mice (Figure 3A (d)). Therefore, partial loss of polarity and appropriate membrane contextual partitioning precedes cell multilayering in *Scrib*-deficient mammary ducts.

Strict developmental control of proliferation and apoptosis is necessary for mammary morphogenesis where high cell turnover rates are observed within TEB structures prior to their remodelling into newly formed ducts [12], [41]–[43]. Recent studies have suggested that loss of *Scrib* can regulate apoptosis to provide a survival advantage within several experimental settings [29], [44], [45]. We speculated that changes in proliferation or apoptosis at a late stage of ductal morphogenesis were responsible for the delayed onset of ductal hyperplasia. Therefore we quantified immunostaining for BrdU and Cleaved Caspase 3 in TEB structures and newly formed ducts of 6 week virgin mice to understand how *Scrib* loss impacted on distinct phases of ductal morphogenesis and homeostasis. No changes in proliferation or apoptosis rates were observed in TEBs, however, a significant and gene-dosage dependent decrease in apoptosis was observed in immature ducts of 6 week old mice that carried one or two mutated alleles of *Scrib* (Figure 3C). Together these data indicate that *Scrib*-deficient mammary epithelial cells fail to respond to apoptotic cues during mammary duct maturation.

Deregulation of polarity and adhesion proteins can lead to misoriented cell divisions. Accordingly, we reasoned that abnormal oriented cell division might contribute to *Scrib*-deficient multilayering. Scribble has been implicated in the control of spindle orientation in *Drosophila* [46], and recent experiments have characterized a role for regulators of polarity, including E-cadherin, Cdc42 and Scribble, in controlling spindle orientation during mammalian symmetric divisions and directed migration [2], [47]–[51]. Because these data indicate a role for Scribble in the control of spindle orientation, we assessed the regulation of planar divisions within the luminal epithelial plane of mammary ducts in wildtype and *Scrib*-deficient mammary glands (6 week virgin mice). This was achieved through immunofluorescence and confocal measurement of spindle geometries prior to the onset of multilayering. Although more divisions are found in peripubertal ducts compared to mature 12 week ducts, the cell divisions within these ducts remain relatively rare in comparison to that commonly observed in TEB structures. Therefore Phospho-Histone H3 (pH 3) was used to locate luminal cells undergoing mitosis whilst pericentrin labelled centrosomes and CK5 labelled the underlying myoepithelial layer to provide a reference for calculation of spindle alignment (Figure 3D (a)). Three-dimensional reconstructions of each division were generated from 18–22 *z*-sections spaced at 0.5 µm intervals (Figure 3D (b)). Divisions that did not contain two centrosomes or those not aligned within the ductal cross section were omitted.

Here we demonstrate that two coordinated cell divisions occur within the luminal layer of the peripubescent mammary duct, those aligned parallel (38% 0^0^–20^0^) to the epithelial sheet and those aligned perpendicular (29% 70^0^–90^0^). Parallel divisions are symmetric and are considered to maintain luminal cell expansion within the epithelial plane, whereas perpendicular divisions represent an as yet uncharacterized division, likely to be asymmetric. Importantly, in the context of *Scrib*-depletion, luminal cell division becomes erratic with almost two-fold less divisions occurring within the epithelial plane (20% 0^0^–20^0^) compared to control mice, associated with a distinct increase in angular cell divisions (Figure 3D (c)). This suggests that Scribble is required to regulate spindle orientations within the mammary ductal epithelium, and that deregulation of division axis may be a contributing factor to the *Scrib*-deficient multi-layering phenotype.

### *Scrib* loss perturbs murine luminal differentiation but not mammary stem cell turnover *in vivo*

**Figure 4:**
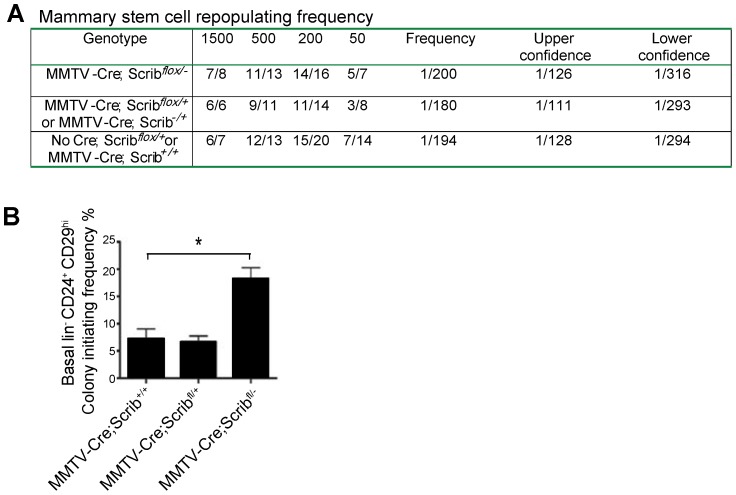
*Scrib* loss impacts on basal progenitors activity but not mammary stem cell turnover *in vivo*. **A.** Limiting dilution analysis of the repopulating frequency of CD29^hi^CD24^+^ cells from *MMTV-Cre;Scrib^flox/−^* and Control mice. Number of positive outgrowths is shown as number of outgrowths per number of mammary fat pads injected. **B.** Colony formation assay measuring increased clonogenic potential of FACS purified lin^−^/CD24^+^/CD29^hi^ basal cell populations from *MMTV-Cre;Scrib^flox/−^* mice grown on irradiated 3T3s. n = 3.

Given the potential role for *Scrib* in asymmetric cell division and spindle orientation, we next examined the mammary gland epithelial cell hierarchy to delineate which epithelial subpopulations were affected by *Scrib* loss and could potentiate the clonal expansion of luminal cells. The basal (CD24^+^CD29^hi^) population is enriched for mammary stem cells (MaSCs), basal progenitors and differentiated myoepithelial cells while the luminal (CD24^+^CD29^lo^) population consists of luminal progenitors and differentiated ductal cells [11], [52]. To further examine the properties of the different epithelial populations we performed fluorescence-activated cell sorting using freshly dissociated mammary tissue isolated from 10 week old virgin mice and conducted a series of specific functional assays. First, to assess the impact of *Scrib* loss on MaSC function, we transplanted limiting numbers of cells from the MaSC-enriched population (CD24^+^CD29^hi^) isolated from three genetic groups which included *MMTV-Cre;Scrib^+/+^* and *Scrib* ^flox/+^ control mice, *MMTV-Cre;Scrib^flox/+^* and *MMTV-Cre;Scrib^+/−^* heterozygous mice and *MMTV-Cre;Scrib^flox/−^* mutant mice into the cleared mammary fat pads of isogenic recipient mice (Figure 4A). No difference in repopulating efficiency was observed between the genetic groups, indicating that *Scrib* loss does not impact on MaSC function or number. We next evaluated whether targeted deletion of *Scrib* impacted on progenitor cells by performing colony assays with basal (CD24^+^CD29^hi^) and luminal progenitor (CD24^+^CD29^l^°CD61^+^) cell populations. A dramatic increase in the clonogenic potential of *Scrib*-deficient CD24^+^CD29^hi^ basal cells was observed on irradiated 3T3 feeder cells (Figure 4B), suggesting an increase in the absolute number of progenitor cell types in this subset. No significant change in the colony forming capacity of *Scrib*-deficient luminal progenitor subset was observed (data not shown). In addition, *Scrib* deficient basal (CD24^+^CD29^hi^) cell populations embedded in Matrigel yielded higher numbers of organoids (Figure S2B & C). The number of basal and surprisingly luminal populations did not significantly vary in *Scrib*-deficient mammary glands compared with controls by flow cytometry analysis. Our observation that intraluminal *Scrib*-deficient cells show increased apoptosis *in vivo* as measured by Cleaved Caspase-3 (Figure 2E), suggests that these cells are primed for cell death and that the intraluminal proportion on *Scrib*-deficient cells are likely lost early in the dissociation procedure. Altogether, these findings indicate that *Scrib* loss alters the clonogenic potential of a distinct mammary progenitor cell population prior to luminal lineage expansion within the epithelial hierarchy suggesting that *Scrib* is required for the regulation of basal to luminal lineage transition and that the ectopic outgrowth of aberrant luminal cells is initiated within the MaSC/basal compartment.

### *Scrib* loss alters the Notch differentiation pathway and drives MAPK hyperactivation to allow inappropriate luminal filling

**Figure 5:**
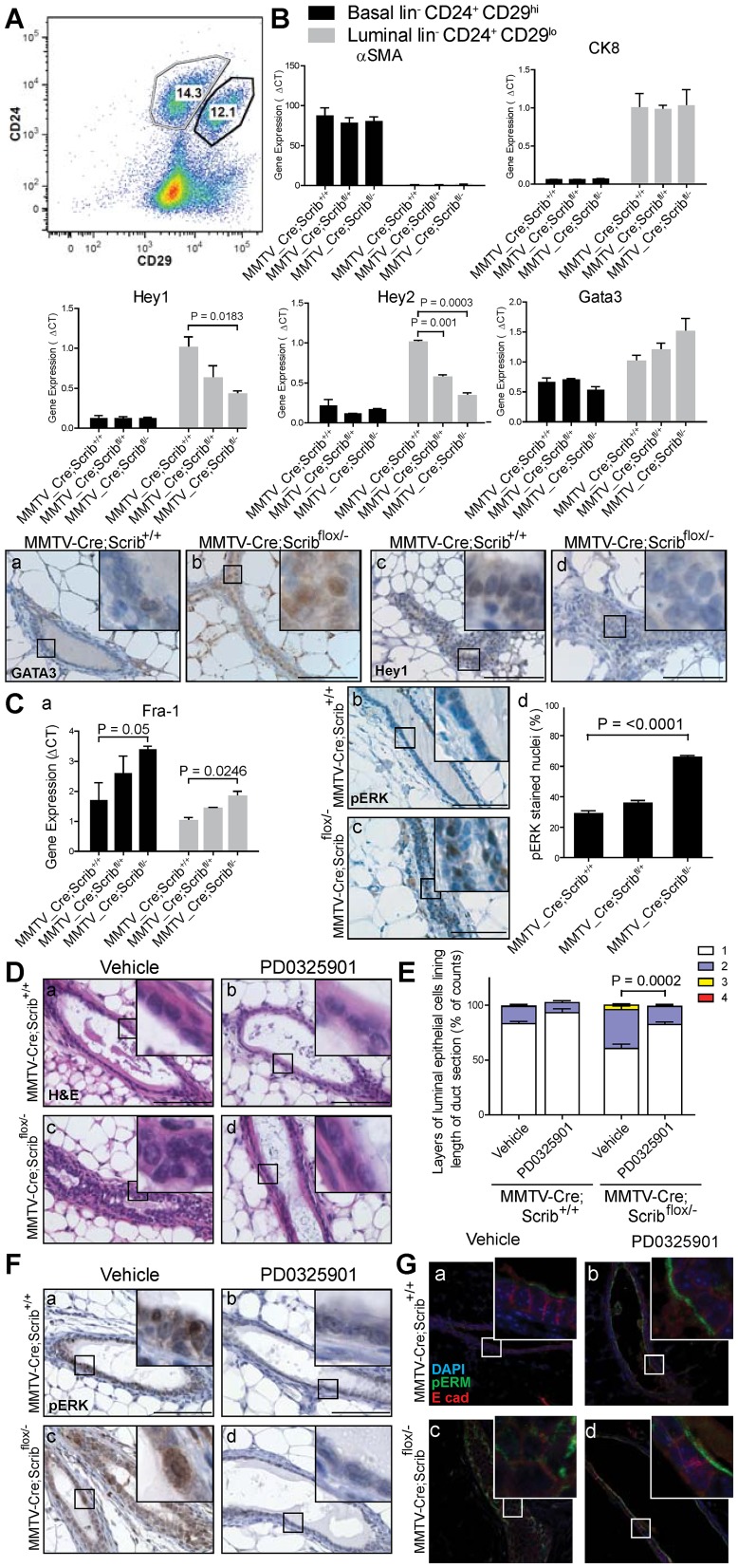
Scribble loss disrupts Notch signalling and drives MAPK/Fra1 activation to allow inappropriate luminal expansion. **A.** Representative FACS scatter plot of lin^−^/CD24^+^/CD29^hi^ basal and lin^−^/CD24^+^/CD29^lo^ luminal cell populations in 8–10 week old Scrib mutant mice. ± SEM. (n = 4–5 per group) **B.** Q-RT-PCR of Notch target genes Hey1 and Hey2, and ductal Gata3 in FACS purified lin^−^/CD24^+^/CD29^hi^ basal and lin^−^/CD24^+^/CD29^lo^ luminal cell populations and IHC confirmation of increased GATA3, and decreased Hey1 staining in hyperplastic ducts of 12 wk *MMTV-Cre;Scrib^flox/−^* virgin mice. Expression levels of luminal maker CK8 and basal marker αSMA confirm purity of cell populations. ± SEM. students t-test, (n = 3, 8–10 week old mice). **C.** Q-RT-PCR of MAPK effector Fra1 in FACS purified lin^−^/CD24^+^/CD29^hi^ basal and lin^−^/CD24^+^/CD29^lo^ luminal cell populations (a), and confirmation of MAPK/ERK pathway activation by IHC (b–d), show significantly increased pERK positive nuclei (unpaired t test with Welch's correction, n = 3–4). Scale bar = 100 µm. **D.** Treatment of 6 week old *MMTV-Cre*, *MMTV-Cre;Scrib^flox/+^* and *MMTV-Cre;Scrib^flox/−^* virgin mice with 20 mg/kg/day PD0325901 5 days on, 2 days off for two weeks blocked the onset of multilayering in *MMTV-Cre;Scrib^flox/−^* mice. Detected by H&E staining (a–d) **E.** Multilayering determined and quantified by counting layers of luminal epithelial cells along length of longitudinal duct H&E sections (i). ± SEM. Mann Whitney t-test, (n = 7–9, 20–39 ducts/mouse). **F.** MAPK inhibition was assessed by IHC of pERK (a–d). Scale bar = 100 µm. **G.** Polarity status by IF of pERM (green) and E-cadherin (red) (a–d). Scale bar = 50 µm. see also Figure S2 and S4, S5.

To determine the molecular pathways by which Scribble may be regulating basal to luminal transition, we examined the impact of *Scrib* deficiency on the gene expression signatures of critical growth and differentiation pathways known to regulate cell fate in the mammary gland including Notch and MAPK. Gene expression analysis was performed on 8 week old *MMTV-Cre;Scrib^+/+^* and *MMTV-Cre;Scrib^flox/−^* mutant mice using unpooled cDNA from freshly sorted basal and luminal mammary epithelial populations (Figure 5A & Procedures S1). The purity of luminal (CD24^+^CD29^lo^) and basal (CD24^+^CD29^hi^) cell populations was confirmed by determining the expression of CK8 and α-smooth muscle actin (α-SMA) by q-RT-PCR analysis (Figure 5B).

The Notch pathway has been shown to be a critical driver of luminal cell fate specification in mammary development with increased gene expression of Notch targets Hey1 and Hey2, but not Hes6, in luminal progenitors compared to mature luminal ductal cells [53]. We observed a significant and dose dependent reduction in Hey1 and Hey2 (but not Hes6) in *Scrib*-deficient luminal cells (Figure 5B and Figure S2D), and an increase in GATA3, a master regulator of luminal lineage differentiation which is highly expressed in mature luminal cells [54] (Figure 5B). Given that duct hyperplasia only occurs in mammary ducts homozygous for *Scrib*-loss and not heterozygous tissue, the gene-dose dependent changes in gene expression in heterozygote tissue indicate they are cell autonomous and do not simply reflect shifts in cell populations. These expression changes were also specific for the luminal compartment as they were not observed within the basal compartment (Figure 5B). We used immunohistochemistry to confirm the suppression of Hey1 and elevation of GATA3 protein levels in *Scrib*-deficient luminal cells in the mammary glands of 12 week old mice (Figure 5B (a–d)). This is consistent with the notion that *Scrib*-deficiency drives the formation of more mature luminal cells rather than progenitor cell types. To assess how *Scrib*-deficiency impacts on the luminal cell program in the mammary epithelium of *MMTV-Cre;Scrib^flox/−^* virgin mice, we next analyzed the mRNA expression of the ductal and alveolar luminal progenitor markers Kit and Elf5 [55], [56]. Similar to the decrease in CD61^+^ cells within the luminal population, Kit and Elf5 expression was moderately decreased in *Scrib*-deficient luminal cells (Figure S2D). To evaluate any impact *Scrib* loss may have during alveolar development, we performed immuno-histological analysis on cohorts of mice during pregnancy (14.5 days post coital & 16.5 days post coital), lactation (4 days post-partum), Involution (day 4 post weaning), and 12 weeks post-pregnancy (Figure S3 & Procedures S1). No histological changes in the ducts and alveoli were apparent, regardless of *Scrib* deficiency. Surprisingly, pregnancy led to rescue of the cell polarity and ductal defects observed in MMTV-*Cre*;*Scrib^flox/−^* mammary glands in the pre-pregnant state (Figure S3B).

Together these data suggest that *Scrib*-loss does not impact on alveolar differentiation, but drives the outgrowth of a more mature but atypical ductal cell type via the well documented Notch differentiation pathway [53].

MAPK plays key roles in different aspects of mammary development and *Scrib* has been characterized as a modulator of the Ras/MAPK pathway previously [25], [31], [37], [57]–[59]. Importantly, expression analysis revealed dose-dependent increases of the MAPK/ERK and MAPK/JNK pathway effector *Fra1* but not the MAPK/JNK effector *c-Jun* in response to *Scrib* loss in both the basal and luminal cell populations (Figure 5Ca and Figure S2D). Fra1 is therefore hyperactivated via the MAPK/ERK pathway in both basal and luminal compartments of *Scrib*-deficient mammary epithelium. This is consistent with *Scrib*-loss modifying basal progenitor function prior to the expansion of aberrant luminal cells. The increased expression of Fra-1, a specific effector of sustained Ras-MAPK signalling [60], [61] in *Scrib*-deficient cells suggests that ectopic activation of Ras/MAPK in this tissue could be contributing to the multilayering phenotype in mammary ducts. To address this, we examined Ras/MAPK signalling in *Scrib*-deficient mammary epithelium. We detected a significant increase in nuclear pERK expression (over two fold increase), in the *Scrib*-deficient ducts of 12 week virgin mice indicating *Scrib* loss results in activation of MAPK in mature mammary ducts *in vivo* (Figure 5C b–d). Although the Akt pathway was recently shown to be enhanced in cell lines following *Scrib* knockdown [62], we could not detect Akt pathway activation in the mouse mammary epithelium *in vivo* (Figure S4).

To ascertain the relative contribution of elevated Ras/MAPK levels to ductal hyperplasia in *Scrib*-deficient ducts, we treated 6 week old mice (prior to the development of hyperplasia) with the MEK inhibitor PD0325901 for two weeks (n = 7–9). Multilayering of mammary ducts in vehicle-treated *MMTV-Cre;Scrib^flox/−^* mice was readily detectable at 8 weeks, although less severe than that in 12 week-old mice (Compare Figure 1E (f) to Figure 5D (c)). Importantly, histological analysis revealed PD0325901 treatment blocked the early onset of multilayering in *Scrib*-deficient mammary ducts compared with vehicle (Figure 5E)(*P = 0.0002*). Treatment with PD0325901 also suppressed the hyperproliferation associated with *Scrib*-loss in the mature duct epithelium (Figure S5A). Effective MEK inhibition by PD0325901 was confirmed by immunostaining for pERK (Figure 5F (a–d)). Strikingly, we also observed a more organized and tightly packed mammary epithelium with restoration of laterally enriched E-cadherin and a clearly defined pERM positive apical membrane region in ducts of PD0325901 treated *MMTV-Cre;Scrib^flox/−^* mice compared to ducts from vehicle treated mice (Figure 5G (a–d)). Thus, hyperactivation of MAPK as a consequence of Scribble loss of function is largely responsible for the loss of cell polarity and tissue organization observed in *Scrib* deficient mammary tissue.

Our findings show that hyperactivation of the Ras/MAPK pathway induced by *Scrib*-loss is necessary to allow the accumulation of intraluminal mammary epithelial cells. Altogether we show that *Scrib*-deficiency and Ras/MAPK pathway activation results in the over growth of unpolarised but mature ductal luminal cells with elevated Fra1 expression.

### *Scrib* loss enhances mammary tumour progression

**Figure 6:**
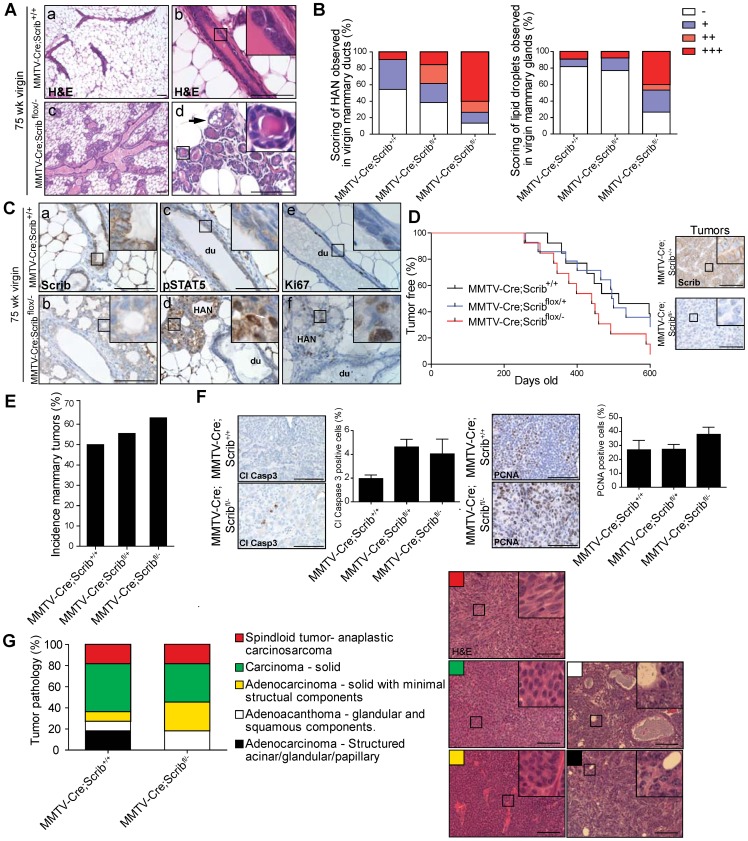
Scribble loss enhances mammary tumourigenesis. **A.** Histological analysis of ductal architecture in 75 week mice by H&E staining show hyperplastic alveolar nodules (HAN) in *MMTV-Cre;Scrib^flox/−^* mice (c, d) compared to control (a, b) as indicated by appearance of circular lobules and lipid droplets (d, black arrow). **B.** Quantitation of rare (<20%, +), frequent (20–80%, ++), or extensive (>80%, +++) observable alveolar or lipid droplets within mammary epithelium. **C.** IHC of Scribble (a, b), pSTAT5 (c, d) and Ki67 (e, f) in mammary ducts (du) or HAN of 75 week *MMTV-Cre* control or *MMTV-Cre;Scrib^flox/−^* mice. Scale bar = 100 µm. **D.** Tumour-free plot for *MMTV-Cre* (n = 19), *MMTV-Cre;Scrib^flox/+^* (n = 20) and *MMTV-Cre;Scrib^flox/−^* (n = 19) virgin mice palpated weekly for mammary tumours. Scribble loss detected by IHC in tumours from *MMTV-Cre;Scrib^flox/−^* aged mice. Scale bar = 100 µm. **E.** Incidence of mammary tumours amongst cohorts of MMTV-Cre, MMTV-Cre;Scribflox/+ and MMTV-Cre;Scribflox/− 525 day old virgin mice. **F.** Tumours from *MMTV-Cre;Scrib^flox/−^* aged mice show increased cell turnover rates as quantified by PCNA and Cleaved caspase 3 IHC. Scale bar = 100 µm. **G.** Comparative tumour pathologies show tumours from *MMTV-Cre;Scrib^flox/−^* aged mice (n = 11) are more progressed and lack structural acinar/glandular characteristics compared to tumours from *MMTV-Cre* mice (n = 11). Representative H&E staining of tumour classifications. Scale bar = 100 µm. see also Figure S6.

Deletion of *Scrib* in the mammary epithelium is characterized by differentiation defects, persistent Ras/MAPK hyperactivation, increased cell turnover, altered directional divisions and compromised tissue integrity. We predicted that these defects coupled with disruption to normal cell polarity signalling would act in concert to promote several aspects of mammary tumourigenesis. We therefore aged cohorts of virgin wildtype, *Scrib^+/−^*, *MMTV-Cre;Scrib^+/+^*, *MMTV-Cre;Scrib^flox/+^* and *MMTV-Cre;Scrib^flox/−^* mice up to 75 weeks. Upon histological examination, we discovered widespread patterns of hyperplastic alveolar nodules (HAN) in the mammary glands of *MMTV-Cre;Scrib^flox/−^* virgin mice (Figure 6A (c–d)). These preneoplastic lesions show increased tumourigenic potential compared to ductal hyperplastic lesions when assayed by transplantation [63]. HAN can be characterized by focal disorganized alveolar ringlets with lipid droplets, increased proliferation and STAT5 hyperphosphorylation [63], [64]. We observed significantly increased regions of HAN and lipid droplets in mammary glands of *MMTV-Cre;Scrib^flox/−^* mutant mice compared to control mammary glands (Figure 6B). These HAN lacked *Scrib* expression and in comparison to adjacent ducts exhibited a dramatic increase in proliferation and hyperactivation of STAT5 as expected (Figure 6C (a–f)).

To determine whether the HAN lesions indicated a greater sensitivity to tumour progression in *Scrib*-deficient mammary tissue, we monitored aged cohorts for tumour occurrence and survival. Although *MMTV-Cre;Scrib^flox/−^* mice showed reduction in survival (475 days) compared to 525 days in *MMTV-Cre* control mice this was not statistically significant (Figure S6A). Many mice exhibited mammary tumours and to a lesser extent ovarian and lung tumours at endpoint. Spontaneous mammary tumours are a common event in FVB/n mice and exhibit a broad spectrum of lesions that encompasses the natural history of mammary tumour progression [65]. We therefore also monitored *MMTV-Cre;Scrib^+/+^*, *MMTV-Cre;Scrib^flox/+^* and *MMTV-Cre;Scrib^flox/−^* mice for mammary tumour onset. Both *MMTV-Cre;Scrib^flox/−^* and *MMTV-Cre;Scrib^flox/+^* mice developed mammary tumours earlier (median tumour initiation at 449 and 492 days respectively) than *MMTV-Cre* control mice (586 days) (Figure 6D). All tumours that arose from *MMTV-Cre;Scrib^flox/−^* mice were shown to have a clear absence of *Scrib* compared to tumours from *MMTV-Cre* mice as determined by IHC (Figure 6D). The incidence of stochastic mammary tumours increased significantly in *MMTV-Cre;Scrib^flox/−^* mice compared to *MMTV-Cre* control mice (Figure 6E). Similar to our earlier observations within *Scrib*-deficient hyperplastic mammary ducts we identified a trend for higher cell turnover in conjunction with *Scrib* loss in mammary lesions (Figure 6F). To understand the impact of *Scrib* loss on the progression of mammary tumourigenesis, we compared the histopathological grading of tumours from *MMTV-Cre;Scrib^+/+^* and *MMTV-Cre;Scrib^flox/−^* mice at endpoint (1200 mm^3^ tumour volume) using a broadly accepted nomenclature and morphologic classification system [66]. Briefly, low grade lesions are characterized by the presence of residual structures such as glandular/acinar patterning, hyperchromatic nuclei and reduced cytoplasm without pleomorphism whereas high grade lesions have less differentiated solid masses of neoplastic cells with nuclear pleomorphism and invasion into adjacent mammary tissue [66]. Tumours from control mice exhibited a similar range of mammary tumour phenotypes to that previously reported, from well-structured adenocarcinomas to solid sheets of carcinoma cells and spindleoid mammary tumours which are reminiscent of an epithelial to mesenchymal transition [65]. We observed an absence of low grade mammary tumours in *MMTV-Cre;Scrib^flox/−^* animals indicating that *Scrib* loss further promotes loss of architecture and differentiation in the context of a tumour (Figure 6G). In addition, we immunostained a subset of these tumours using the basal-like tumour marker CK14 and found more tumours from *MMTV-Cre;Scrib^flox/−^* mice stained positive for CK14 expression (4/5), compared to those from control mice (0/5)(Figure S6B). Overall, tumours from *MMTV-Cre;Scrib^flox/−^* mice size and age matched to low grade control tumours were either spindleoid EMT type or solid carcinomas. Therefore loss of Scribble function not only replicates the most initial phase of pre-malignancy in the breast but can accelerate tumour progression and promote the development of basal-like less differentiated tumours.

Taken together these data have not only defined a distinct role for *Scrib* in mammary duct morphogenesis, luminal differentiation and tissue homeostasis *in vivo*, but also demonstrates that *Scrib* tumour suppressor function is a critical modulator of multiple steps in mammary tumourigenesis from premalignancy to the evolution and progression of sporadic mammary tumours.

## Discussion

### Role of cell polarity in breast development

We have demonstrated a requirement for *Scrib* in the homeostasis of the mammary ductal luminal epithelium. In particular, we show that *Scrib* plays an essential role mediating the correct modelling of newly formed mammary ducts during the final phase of duct morphogenesis, a process that involves the clearance of apoptotic cells and the maintenance of multiple aspects of cell polarity, including the resolution of apical and basolateral identity and correct spindle positioning. Whilst the initial stages of mammary duct formation are well characterized and are known to involve high proliferation and collective movements of depolarized cells within the TEB followed by apoptosis [12], [41], much less is known of any late stage or redundant mechanisms regulating mammary lumen formation and maintenance. One example of this involves the pro-apoptotic protein BIM, a key driver of apoptosis during lumen formation *in vitro* and *in vivo* [67], [68]. BIM loss in the mouse mammary gland results in defects in apoptosis and lumen clearing resulting in multilayered ducts during puberty. These ducts bear remarkable morphological similarity to *Scrib*-deficient ducts however the multilayering arises directly from the TEB and is rescued by unknown late stage mechanisms after 5 weeks of age [67]. WAP-Bcl-2 transgenic mice exhibit a similar delay in lumen formation [41]. These findings suggest a late-phase of duct maturation occurs which utilizes additional mechanisms of tubulogenesis. A role for lumen formation via remodelling has been demonstrated in Matrigel cultures of primary mammary epithelial cells [69] and has been proposed previously [3], [42], [43]. Such a phase of duct maturation likely involves residual apoptosis, proliferation, cell movements and the reinforcement of apical-basal polarization in newly formed mammary ducts prior to a more quiescent stage at maturity. Our findings support this model and implicate cell polarity control as an essential driver of mammary duct maturation *in vivo*. We show loss of polarity control does not impair lumen formation at the TEB, but results in insufficient apical-basal polarization, aberrantly orientated cell divisions, sustained activation of the MAPK pathway and the evasion of a late phase of apoptosis associated with duct maturation. These findings confirm and extend the observations by Mailleux, et. al. (2007) and Humphreys, et. al. (1996), that in addition to apoptosis, control of cell polarity is one of the key mechanisms required for duct formation.

Interestingly, *Scrib* loss results in several morphological defects in the mouse mammary epithelium which cannot be recapitulated by growing the same *Scrib* deficient mammary epithelial cells in Matrigel cultures (Figure S2). Luminal-myoepithelial cell and epithelial-stromal interactions are often absent in 3D cultures and epithelial cells are instead saturated by growth factor signals and basement membrane contextual cues which we suggest override polarity defects. These findings highlight the need to use appropriate mouse models to definitively describe the role of polarity genes in mammary morphogenesis.

In addition to the influence of the ECM on cell polarity defects, we found *Scrib* modulation of the Ras/MAPK pathway to be critical in maintaining epithelial tissue polarity, as inhibition of the Ras/MAPK was able to prevent the onset of duct hyperplasia and restore apical-basal polarity. Altogether these observations suggest that intact tissue polarity cues can correct defects in core cell polarity signalling in some contexts.

Previously *Scrib* has been implicated in the control of mitotic spindle asymmetry during Drosophila neuroblast divisions [46]. We show for the first time that *Scrib* coordinates spindle orientation in a mammalian developmental context *in vivo*. We have characterized the distribution of mitotic spindle angles in the luminal layer of the mammary duct *in vivo* and demonstrate a requirement for *Scrib* in the regulation of distinct parallel and perpendicular divisions of luminal epithelial cells. Loss of *Scrib* leads to deregulation of spindle positioning during planar divisions which can give rise to a multilayered epithelium. Indeed mitotic spindle misorientation has been proposed to contribute to several aspects of cancer progression [70]. Supporting our studies of spindle orientations in the luminal layer of *Scrib*-deficient ducts and the mammary duct branching defects observed in these mice, mice lacking β1 integrin in basal cells exhibit similar spindle orientation defects coupled with an abnormal ductal branching pattern [71]. This suggests spindle orientation defects may also impact on branching morphogenesis and that in addition to polarity mechanisms, cell-ECM interactions also play an important role in directing spindle orientations in mammary ducts. Interestingly, modulation of the Ras/MAPK pathway itself can control spindle orientation during lung branching morphogenesis to drive epithelial tube morphogenesis [72]. It is therefore likely a similar process exists in the developing mammary gland. Indeed *Scrib* loss results in Ras/MAPK signalling, spindle orientation and branching morphogenesis defects. Further studies will be required to elucidate the relationship between Rash/MAPK pathway and spindle orientation and branching in the mammary gland.

### Scribble is a critical modulator of MAPK activity during breast development and homeostasis

**Figure 7:**
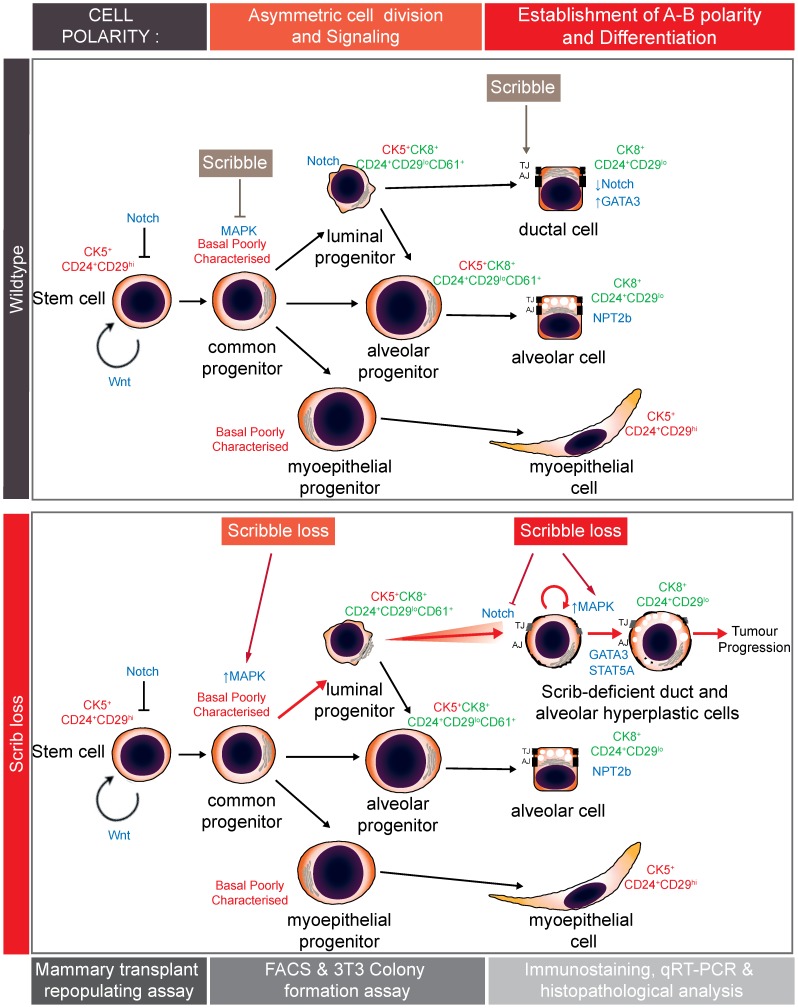
Model summarizing impacts of Scribble in mammary development and homeostasis. In normal mammary development, Scribble moderates basal progenitor clonogenic potential and controls spindle orientation to maintain a balanced epithelial hierarchy. Scribble is also required for remodelling and to establish apical-basal polarity during late stage duct morphogenesis. Scribble loss leads to hyperactivation of the Ras/MAPK pathway and the expansion of ectopic luminal epithelial cells to promote tumour development.

The Ras/MAPK pathway is strongly implicated in morphogenesis of branched epithelial tissues such as salivary gland [73], kidney [74], and lung [72], but less is known about the role of Ras/MAPK flux in mammary morphogenesis. We now reveal that modulation of MAPK by cell polarity mechanisms is required during mammary development, specifically to limit the activity of basal progenitors and to allow the terminal differentiation and homeostasis of the ductal luminal epithelium (Figure 7).

We observed dose-dependent increases in Fra1 expression associated with *Scrib* loss in both basal and luminal mammary epithelial cell populations. This suggests a critical role for *Scrib* suppression of Ras/MAPK within the mammary cell hierarchy and is consistent with our findings *in vitro* where Scribble knock down elicits a heightened and sustained MAPK response concomitant with elevated Fra1 [37]. Fra1 levels are strongly associated with sustained Erk1/2 signalling kinetics [75], [76], and sustained compared to transient MAPK/ERK responses drive different morphological responses in mammary epithelial cells. In cultures of primary mouse mammary organoids, sustained MAPK/ERK activation elicits branching morphogenesis and an expansion of cells positive for K6, a marker of hyperproliferative progenitor cells, whereas transient MAPK/ERK activation elicits growth without branching [77]. More recently, human organoids in culture were treated with EGFR ligands AREG or EGF. Both ligands induced ductal outgrowth however EGF, which results in a more sustained MAPK/ERK activation [78], resulted in the initial expansion of dual-positive K8+/K14+ progenitor cells followed by ectopic expansion of myoepithelial cells [79]. These findings suggest differences in MAPK/ERK signalling kinetics impact on duct morphogenesis and lineage specification. Previous studies investigating the distinct roles of growth factors and ErbB receptors during mammary development also support the notion that different MAPK kinetics result in alternate differentiation outcomes [58], [80].

We have demonstrated that polarity control is a key modulator of MAPK flux during mammary duct morphogenesis as *Scrib*-deficient mature mammary ducts exhibit ectopic hyperactivation of the Ras/MAPK/Fra1 pathway. A number of studies have shown persistent Ras/MAPK pathway activation undermines mammary epithelial organization. Transformed mammary epithelial cells fail to form lumens in Matrigel cultures [81]. Such breast cancer cell lines exhibit persistent Ras/MAPK activation and suppression of the Ras/MAPK pathway and β1 integrin inhibition induces phenotypic reversion [82]. Furthermore, oncogenes that act on the Ras/MAPK pathway disrupt apical-basal polarity in epithelial organoids [83]–[85]. Activation of ErbB2 in mice can result in HAN similar to those that develop in *MMTV-Cre;Scrib^flox/−^* mice and conversely the specific deletion of ErbB2 impairs duct elongation and branching morphogenesis [86], [87]. Altogether these findings suggest that deregulation of normal MAPK and cell polarity signalling are concomitant processes in mammary tumourgenesis.

We have now characterized Scribble as a modifier of MAPK/ERK signalling kinetics in mammary cell line models [31], [37] and in multiple tissues *in vivo* [25], [57]. Our mouse model of *Scrib* loss will therefore be an important tool to understand the impact of sustained Ras/MAPK activation in mammary ducts *in vivo*.

### Disruption of cell polarity can contribute to the initiation and progression of breast cancer

Multilayering, loss of polarity and luminal filling can define premalignant breast lesions such as breast ductal hyperplasia and DCIS. However the molecular mechanisms that initiate these events are unknown. In addition to polarity loss, oncogenes that drive proliferation and survival such as HER-2, Cyclin D1 and C-MYC are frequently activated in pre-invasive breast lesions of high malignant potential such as DCIS and allow survival in the luminal space [88], [89]. We have found that disruption to the core polarity network and activation of MAPK may participate together in early events prior to DCIS and breast cancer development. We have recently found that many polarity genes suppress sustained activation of MAPK (Smith et al, manuscript in preparation), and deregulation of *Scrib* in human breast cancer has been reported previously [27]–[29]. Furthermore, several common molecular lesions in DCIS promote Ras/MAPK signalling [90]–[93]. In particular, Fra1 is highly expressed in breast ductal hyperplasia and DCIS and is therefore associated with early events in breast carcinogenesis [94]–[97]. Therefore, disruption of tissue polarity, high cell turnover and hyperactivation of a MAPK/Fra1 oncogenic pathway is likely to lead to a more rapid accumulation of genetic abnormalities and escalate mammary tumour progression. Accordingly, *Scrib* loss leads to the increased incidence of mammary tumours which are less histologically differentiated. Poorly differentiated human breast tumours are characterized by a loss of polarity, elevated Fra1 levels and worse outcomes [96], [98], [99].

We have also observed that pregnancy can rescue the *Scrib*-hyperplastic phenotype and associated polarity defects. In humans, cancer risk is dramatically decreased with early pregnancy [100]. Our observations support the notion that pregnancy is likely to drive the alveolar differentiation of uncommitted luminal epithelial cells which may be more prone to tumourigenesis [101].

We have used a definitive conditional mouse model of *Scrib*-loss in the mammary gland to show a requirement for *Scrib* in the control of basal progenitor function and luminal cell maturation during mammary duct morphogenesis. Our findings provide new insights into how loss of polarity impacts on tissue homeostasis and can contribute to the etiology and pathogenesis of breast cancer.

## Materials and Methods

### Experimental animals

Both null and conditional floxed mutant alleles of *Scrib* were generated in mice as described previously [25].The *Scrib* null allele was produced by crossing *Scrib^flox/+^* mice to a germline Cre-deleter mouse. To generate MMTV_Cre;*Scrib^flox/−^* mice, we crossed female heterozygous *Scrib* null mice to male.

Tg(*MMTV-cre*)1Mam (line A) mice were a kind gift from K Wagner [33]. Male MMTV_Cre;*Scrib^+/−^* mice were then finally crossed to female *Scrib^flox/+^* mice to produce experimental cohorts. Mice were genotyped from genomic DNA derived from toe biopsies using primers for Scrib alleles and conditions as described previously [25].

### Ethics statement

All mice were maintained on a pure FVB/n background (Backcrosses onto pure FVB/n, N>10). All animal use was approved by the Peter MacCallum Cancer Centre Animal Experimental Ethics Committee Ethics Committee and in compliance with National Health and Medical Research Council (Australia) guidelines.

### PD0325901 administration

The treatment regime was similar to that previously reported [25] where MEK inhibitor PD0325901 (JS Research Chemicals Trading) was dissolved in 0.5% hydroxypropyl-methylcellulose; 0.2% Tween 80 at 8 mg/ml and delivered by oral gavage at 20 mg/kg 5 days on and 2 days off from 6 to 8 weeks of age. Mice were weighed daily to monitor drug tolerance and showed no significant reduction in weight (>10% loss).

### Whole mount, immunohistochemistry and immunoblotting analysis

Inguinal mammary fat pads were fixed overnight in Carnoy's fixative solution and immersed in Acetone for at least 3×2 hr before hydration from 70% ethanol. Whole mounts were then stained in carmine alumn overnight, dehydrated through series of ethanol solutions and cleared in Xylene followed by Methyl Salicylate. Analysis of ductal trees was performed by scanning entire slides using an Olympus IX81 microscope and counting distal TEBs or measuring the distance between branch points (>50 measurements/mouse whole mount, 3–6 mice/experimental group). For Histology, formalin-fixed paraffin-embedded (FFPE) sections were stained with H&E and assessed by a trained pathologist. For IF or IHC staining, antigen retrieval was performed on frozen or FFPE sections of inguinal mammary fat pads (>3 mice/experimental group) in 1× citrate buffer (Thermo Scientific) or Tris-EDTA pH 9 (DAKO) in a pressure cooker (125°C 15 psi). IHC samples were blocked in 2% BSA in TBST solution and endogenous peroxidise activity was inactivated in 1.5% H_2_O_2_. Biotinylated species specific secondary antibodies were used at 1∶300 (DAKO) followed by the Avidin Biotin Complex (ABC) Method prior to visualisation with 3,3- Diaminobenzidine chromagen (DAKO). IF samples were blocked in 1X PBS/5% normal donkey serum/0.3% Triton X-100 and Alexa Fluor antibodies (Invitrogen) where used at 1∶300 before mounting in ProLong Gold with DAPI (Invitrogen). Primary antibodies other than those recently reported [25], included Caspase 3 (9661, Cell Signalling Technology), Brdu (347580, BD Biosciences), p-Histone 3 (641001, Biolegend), pericentrin (ab64448, Abcam), Cytokeratin 5 (PRB-160P, covance), Cytokeratin 8 (ab14053, Abcam), β-catenin (610153, BD Biosciences), GATA3 (sc-268 SantaCruz), Hey1 (ab22614, Abcam), p-STAT5 (9331, Cell Signalling Technologies), ZO-1 (clone R40.76, Millipore). Additional antibodies used for Supplemental data see Procedures S1. Wholemount analysis and IHC scoring was carried out using MetaMorph 6.3 software (Molecular Devices). Immunofluorescence and bright field microscopy was performed on an Olympus BX-51 microscope. Immuno blotting was performed on mammary tissue snap frozen in liquid nitrogen as described [54] using anti-Scribble (clone 7C6.D10) [102], α-tubulin (T5168, Sigma).

### Confocal microscopy and analysis of spindle orientation in ductal epithelium

Confocal microscopy was performed on a NIKON eclipse 90i C2 confocal microscope. For spindle analysis, dividing cells were identified by pHistone3 immunostaining and 18–22x 0.5 µm z-section stacks where captured with 60× objective at 2.5× zoom. 3D reconstructions were assessed using NIS-Elements software and evaluated as outlined in results section.

### Mammary epithelial cell isolation cell sorting and mammary epithelial cell assays

Isolation and cell sorting of primary mammary epithelial cell populations was performed on 8–10 week old mice (n = 6) using fluorescently conjugated antibodies to CD45, TER119, CD31, CD29, CD24, CD61 (BD Biosciences) using a FACS DIVA or FACS Aria [52], [54]. Mammary stem cell limiting dilution reconstitution assays and colony formation assays were also as described [52], [54].

### Gene expression analysis

RNA was isolated and reverse transcribed from freshly prepared FACS sorted primary mammary epithelial cell populations using TRizol and superscript III reagents (Invitrogen). cDNA amplification was performed using the stepOnePlus Real-Time PCR system (Applied Biosystems) Samples were normalised to GAPDH and expression levels calculated using the 2^−dCt^ method [103]. Real-time primers listed in Procedures S1.

## Supporting Information

Figure S1 Heterozygosity does not alter mammary development whereas *Scrib* loss disrupts apical polarisation without impacting on junction formation. **A.** H&E staining show normal lumen formation and bi-layer of luminal and myoepithelial cells in ducts of 12 wk virgin *MMTV-Cre* and *MMTV-Cre*; *Scrib^flox/+^* mice heterozygous for Scribble loss (a–d). Scale bar = 100 µm. **B.** Immunofluorescence in mammary ducts of 12 week mice show normal distribution of luminal (Cytokeratin 8, green), and basal (Cytokeratin 14, red) cell populations in ducts from *MMTV-Cre* mice, whereas an expansion of luminal cells is observed in ducts from *MMTV-Cre;Scrib^flox/−^* mice. Scale bar 20 µm. **C.** Immunofluorescence to detect tight junction protein ZO-1. Scale bar = 50 µm. **D.** Ultrastructural organization and integrity of Tight junction (TJ) and Desmosome (Des) complexes in MMTV-Cre control and MMTV-Cre;Scrib^flox/−^ mice. **E.** Immunoblotting of mammary epithelial tissue lysates show heterozygous or homozygous ablation of Scribble in the mammary gland and E-cadherin and β-catenin protein expression. **F.** IHC of apical membrane marker MUC-1 highlighting extensive disruption to apical membrane specification in ducts of *MMTV-Cre;Scrib^flox/−^* mice compared to control. Scale bar = 100 µm.
(TIF)

Figure S2 Colony formation of *Scrib*-deficient mammary epithelial cells in 3D Matrigel. **A.** Representative FACS scatter plot and quantitation of lin−/CD24+/CD29hi basal and lin−/CD24+/CD29lo luminal cell populations in 8–10 week old *Scrib* mutant mice. ± SEM. (n = 4–5 per group) **B.** Colony formation assay measuring increased clonogenic potential of FACS purified lin^−^/CD24^+^/CD29^hi^ basal cell populations from *MMTV-Cre;Scrib^flox/−^* mice grown in Matrigel. n = 3. **C.** Bright field images of Matrigel cultures of primary mammary cells from MMTV-Cre control and MMTV-Cre;Scrib^flox/−^ mice result in normal monolayered and polarised acini structures. *Scrib* loss confirmed by IHC and acinar polarity by IF for pERM (green), Ecadherin (red) and Scrib (blue). Scale bar = 100 µm. **D.** q-RT-PCR of MAPK effector c-Jun, Notch target gene Hes6 and alveolar differentiation markers, Elf5 and Kit in FACS purified lin^−^/CD24^+^/CD29^hi^ basal and lin^−^/CD24^+^/CD29^lo^ luminal cell populations. Expression levels of luminal maker CK8 and basal marker αSMA confirm purity of cell populations. ± SEM. students t-test, (n = 3, 8–10 week old mice).
(TIF)

Figure S3 Alveolar morphogenesis rescues *Scrib*-hyperplasia and loss of tissue polarity. **A.** Histological analysis of lobuloalveolar architecture by H&E staining in mammary glands during day14.5 and 16.5 pregnancy and day 4 of lactation show rescue of tissue disorganization in alveolae of *MMTV-Cre;Scrib^flox/−^* mice. IHC confirms absence of Scrib in mammary epithelium of pregnant and lactating mice. Scale bar = 100 µm. **B.** Immunofluorescence of E-cadherin (green), Cytokeratin 5 (red) and DAPI staining (blue) in mammary glands shows restoration of lateral E-cadherin membrane staining in mature alveolae of *MMTV-Cre;Scrib^flox/−^* mice. Scale bar = 10 µm. **C.** Mammary function by average litter weights 6–18 days post-partum from wildtype, *MMTV-Cre*, *MMTV-Cre;Scrib^flox/+^* and *MMTV-Cre;Scrib^flox/−^* mothers. Recorded from litters of 7–12 pups. ± SEM. (n = 3–4). **D.** H&E and TUNEL staining and quantitation of involuting mammary glands from *MMTV-Cre*, *MMTV-Cre;Scrib^flox/+^* and *MMTV-Cre;Scrib^flox/−^* mice day 4 post-weening. n = 3.
(TIF)

Figure S4 Akt pathway activity in Scrib deficient mouse mammary epithelium. IHC of pAkt (473), pPRAS40, pS6 show activation of Akt pathway in control samples but not normal or *Scrib*-deficient mouse mammary epithelium. Controls included tumors and mammary glands from MMTV-Cre;Pic3ca^H1047R^ mice, xenograft tumors of PC3 and Ca1h cells and lymph nodes from the Eμ-myc mouse model. Scale bar = 100 µm.
(TIF)

Figure S5 Inhibition of the MAPK pathway. Effective treatment of 6 week old *MMTV-Cre*, *MMTV-Cre;Scrib^flox/+^* and *MMTV-Cre;Scrib^flox/−^* virgin mice with 20 mg/kg/day PD0325901 5 days on, 2 days off for two weeks was determined by inhibition of hyperproliferation. n = 3.
(TIF)

Figure S6 Survival analysis and tumour immunostaining in aged mice. **A.** Kaplan-Meir survival analysis for aged cohorts of *MMTV-Cre* (n = 24) versus *MMTV-Cre;Scrib^flox/+^* (n = 18) and *MMTV-Cre;Scrib^flox/−^* (n = 19) virgin mice. Mice predominantly develop mammary tumors but also succumb to lung and ovarian tumors. **B.** Representative images of immunostaining of basal marker CK14 and luminal marker CK18 in tumors from *MMTV-Cre* and *MMTV-Cre;Scrib^flox/−^* mice.
(TIF)

Movie S1 3D reconstruction from confocal z-series of apical membrane marker pERM (green) and E-cadherin (red) showing normal polarised bilayered epithelium in mammary ducts of 12 week *MMTV-Cre;Scrib^+/+^* virgin mice. Scale bar = 50 µm.
(AVI)

Movie S2 3D reconstruction from confocal z-series of apical membrane marker pERM (green) and E-cadherin (red) showing loss of polarity and tissue disorganisation in mammary ducts of 12 week *MMTV-Cre;Scrib^flox/−^* virgin mice. Scale bar = 50 µm.
(AVI)

Procedures S1 Experimental procedures for developmental staging, ultrastructural analysis, gene expression analysis and immunostaining.
(DOCX)
