# Supplementary figures and images for: Scribble Modulates the MAPK/Fra1 Pathway to Disrupt Luminal and Ductal Integrity and Suppress Tumour Formation in the Mammary Gland
Source: PLoS Genet. 2014 May 22;10(5):e1004323. doi: 10.1371/journal.pgen.1004323 (PMC4031063; doi:10.1371/journal.pgen.1004323)

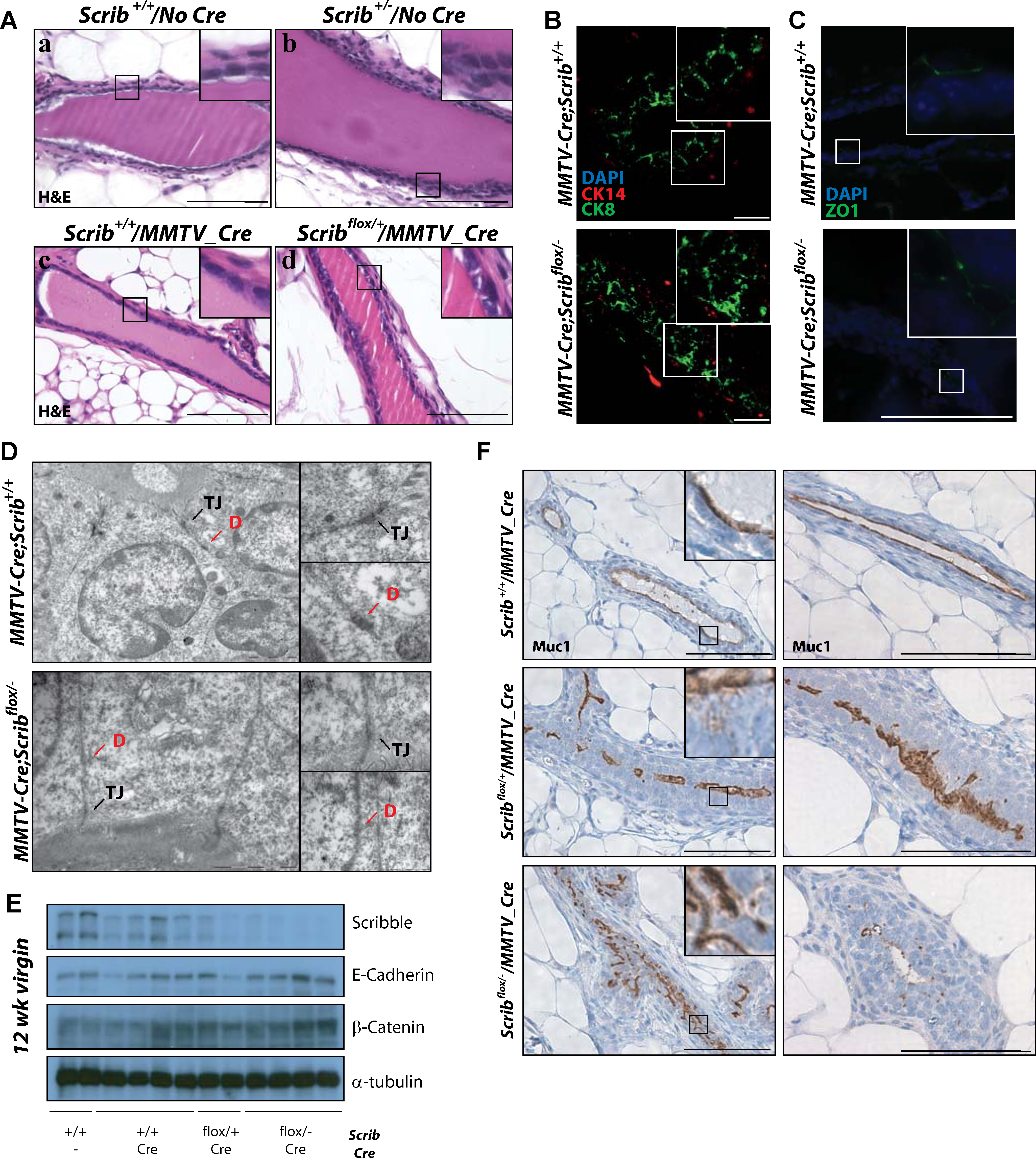

Supplement: Figure S1 — Heterozygosity does not alter mammary development whereas Scrib loss disrupts apical polarisation without impacting on junction formation. A. H&E staining show normal lumen formation and bi-layer of luminal and myoepithelial cells in ducts of 12 wk virgin MMTV-Cre and MMTV-Cre; Scribflox/+ mice heterozygous for Scribble loss (a–d). Scale bar = 100 µm. B. Immunofluorescence in mammary ducts of 12 week mice show normal distribution of luminal (Cytokeratin 8, green), and basal (Cytokeratin 14, red) cell populations in ducts from MMTV-Cre mice, whereas an expansion of luminal cells is observed in ducts from MMTV-Cre;Scribflox/− mice. Scale bar 20 µm. C. Immunofluorescence to detect tight junction protein ZO-1. Scale bar = 50 µm. D. Ultrastructural organization and integrity of Tight junction (TJ) and Desmosome (Des) complexes in MMTV-Cre control and MMTV-Cre;Scribflox/− mice. E. Immunoblotting of mammary epithelial tissue lysates show heterozygous or homozygous ablation of Scribble in the mammary gland and E-cadherin and β-catenin protein expression. F. IHC of apical membrane marker MUC-1 highlighting extensive disruption to apical membrane specification in ducts of MMTV-Cre;Scribflox/− mice compared to control. Scale bar = 100 µm. (TIF) [file pgen.1004323.s001.tif]

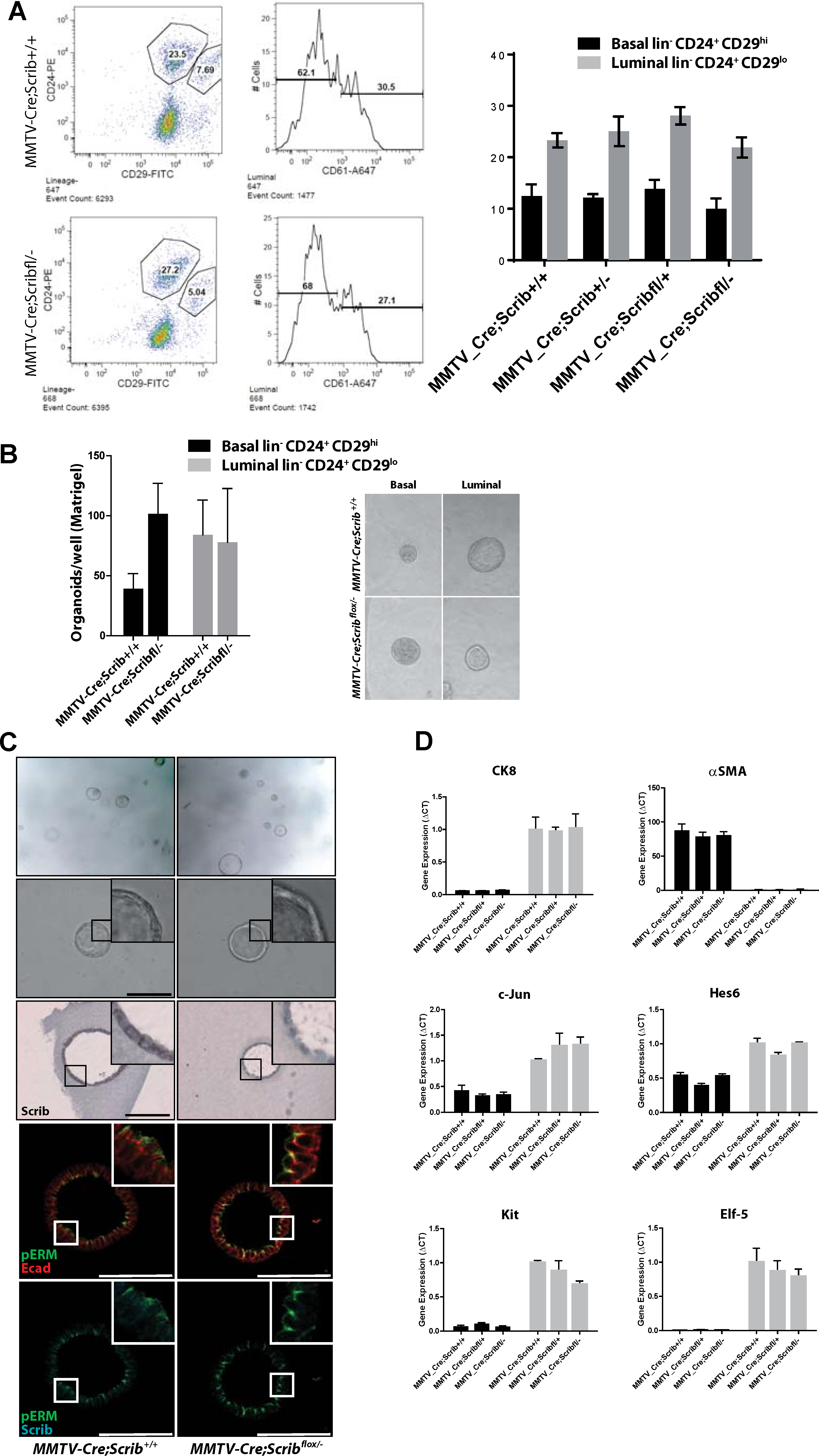

Supplement: Figure S2 — Colony formation of Scrib-deficient mammary epithelial cells in 3D Matrigel. A. Representative FACS scatter plot and quantitation of lin−/CD24+/CD29hi basal and lin−/CD24+/CD29lo luminal cell populations in 8–10 week old Scrib mutant mice. ± SEM. (n = 4–5 per group) B. Colony formation assay measuring increased clonogenic potential of FACS purified lin−/CD24+/CD29hi basal cell populations from MMTV-Cre;Scribflox/− mice grown in Matrigel. n = 3. C. Bright field images of Matrigel cultures of primary mammary cells from MMTV-Cre control and MMTV-Cre;Scribflox/− mice result in normal monolayered and polarised acini structures. Scrib loss confirmed by IHC and acinar polarity by IF for pERM (green), Ecadherin (red) and Scrib (blue). Scale bar = 100 µm. D. q-RT-PCR of MAPK effector c-Jun, Notch target gene Hes6 and alveolar differentiation markers, Elf5 and Kit in FACS purified lin−/CD24+/CD29hi basal and lin−/CD24+/CD29lo luminal cell populations. Expression levels of luminal maker CK8 and basal marker αSMA confirm purity of cell populations. ± SEM. students t-test, (n = 3, 8–10 week old mice). (TIF) [file pgen.1004323.s002.tif]

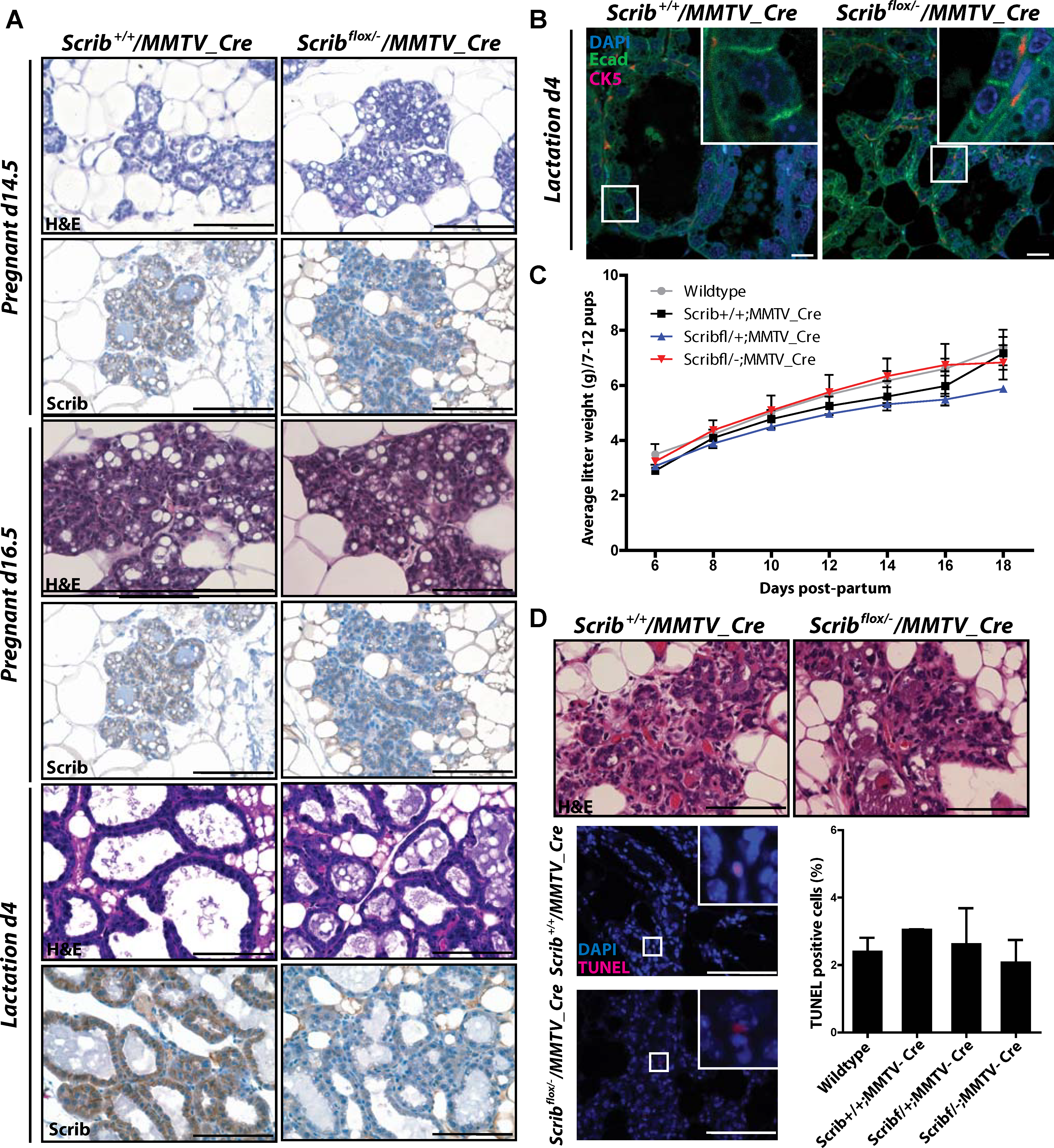

Supplement: Figure S3 — Alveolar morphogenesis rescues Scrib-hyperplasia and loss of tissue polarity. A. Histological analysis of lobuloalveolar architecture by H&E staining in mammary glands during day14.5 and 16.5 pregnancy and day 4 of lactation show rescue of tissue disorganization in alveolae of MMTV-Cre;Scribflox/− mice. IHC confirms absence of Scrib in mammary epithelium of pregnant and lactating mice. Scale bar = 100 µm. B. Immunofluorescence of E-cadherin (green), Cytokeratin 5 (red) and DAPI staining (blue) in mammary glands shows restoration of lateral E-cadherin membrane staining in mature alveolae of MMTV-Cre;Scribflox/− mice. Scale bar = 10 µm. C. Mammary function by average litter weights 6–18 days post-partum from wildtype, MMTV-Cre, MMTV-Cre;Scribflox/+ and MMTV-Cre;Scribflox/− mothers. Recorded from litters of 7–12 pups. ± SEM. (n = 3–4). D. H&E and TUNEL staining and quantitation of involuting mammary glands from MMTV-Cre, MMTV-Cre;Scribflox/+ and MMTV-Cre;Scribflox/− mice day 4 post-weening. n = 3. (TIF) [file pgen.1004323.s003.tif]

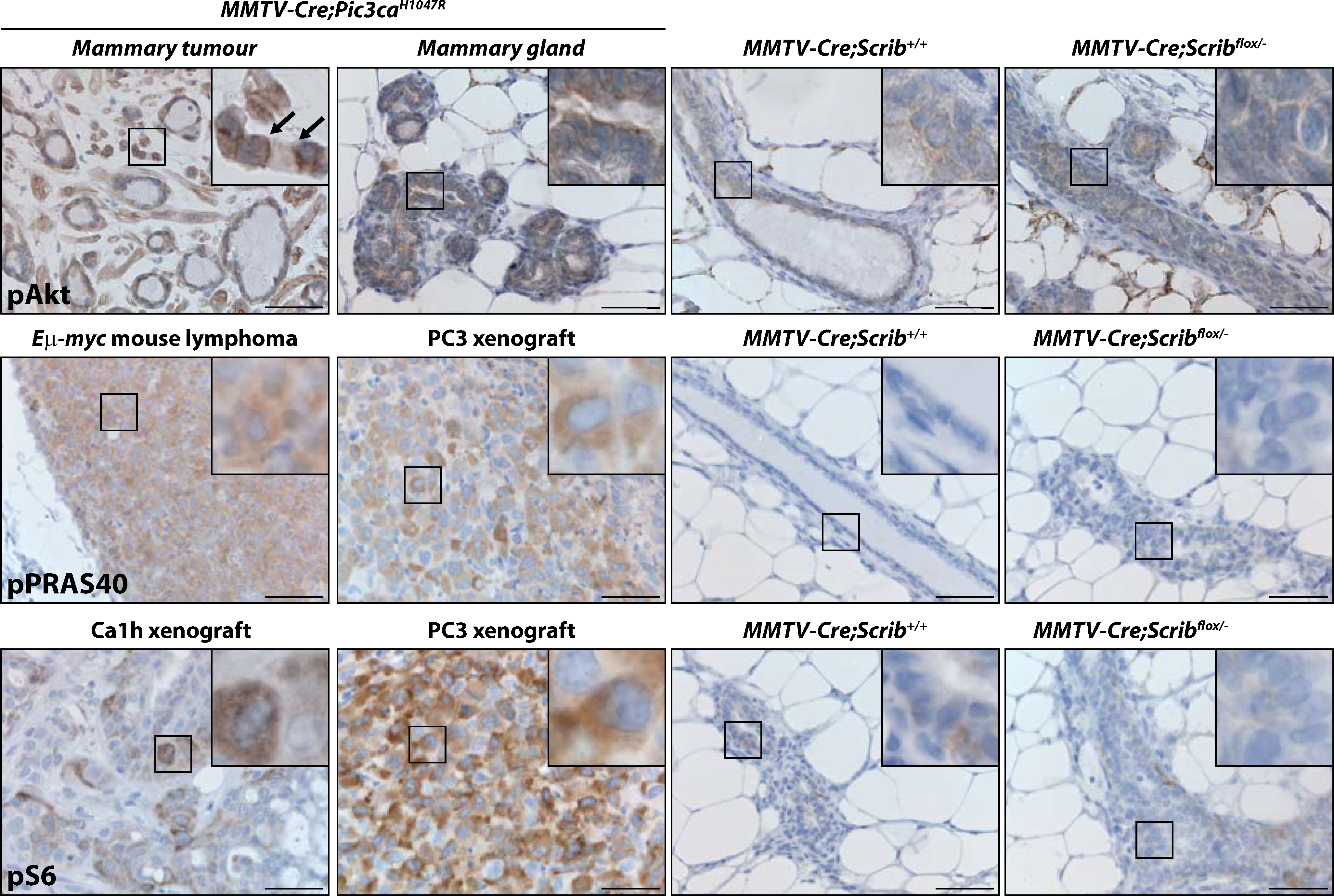

Supplement: Figure S4 — Akt pathway activity in Scrib deficient mouse mammary epithelium. IHC of pAkt (473), pPRAS40, pS6 show activation of Akt pathway in control samples but not normal or Scrib-deficient mouse mammary epithelium. Controls included tumors and mammary glands from MMTV-Cre;Pic3caH1047R mice, xenograft tumors of PC3 and Ca1h cells and lymph nodes from the Eμ-myc mouse model. Scale bar = 100 µm. (TIF) [file pgen.1004323.s004.tif]

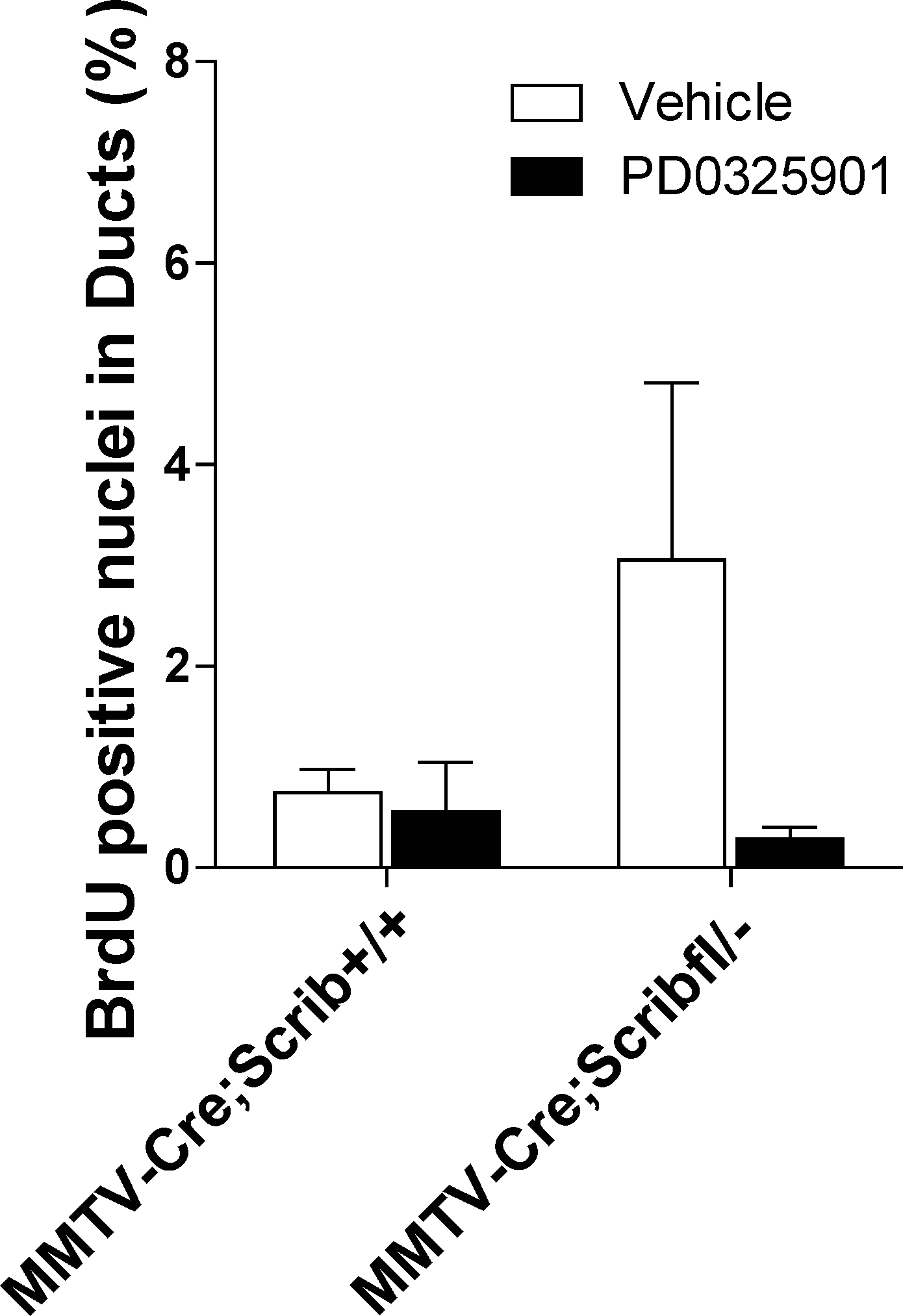

Supplement: Figure S5 — Inhibition of the MAPK pathway. Effective treatment of 6 week old MMTV-Cre, MMTV-Cre;Scribflox/+ and MMTV-Cre;Scribflox/− virgin mice with 20 mg/kg/day PD0325901 5 days on, 2 days off for two weeks was determined by inhibition of hyperproliferation. n = 3. (TIF) [file pgen.1004323.s005.tif]

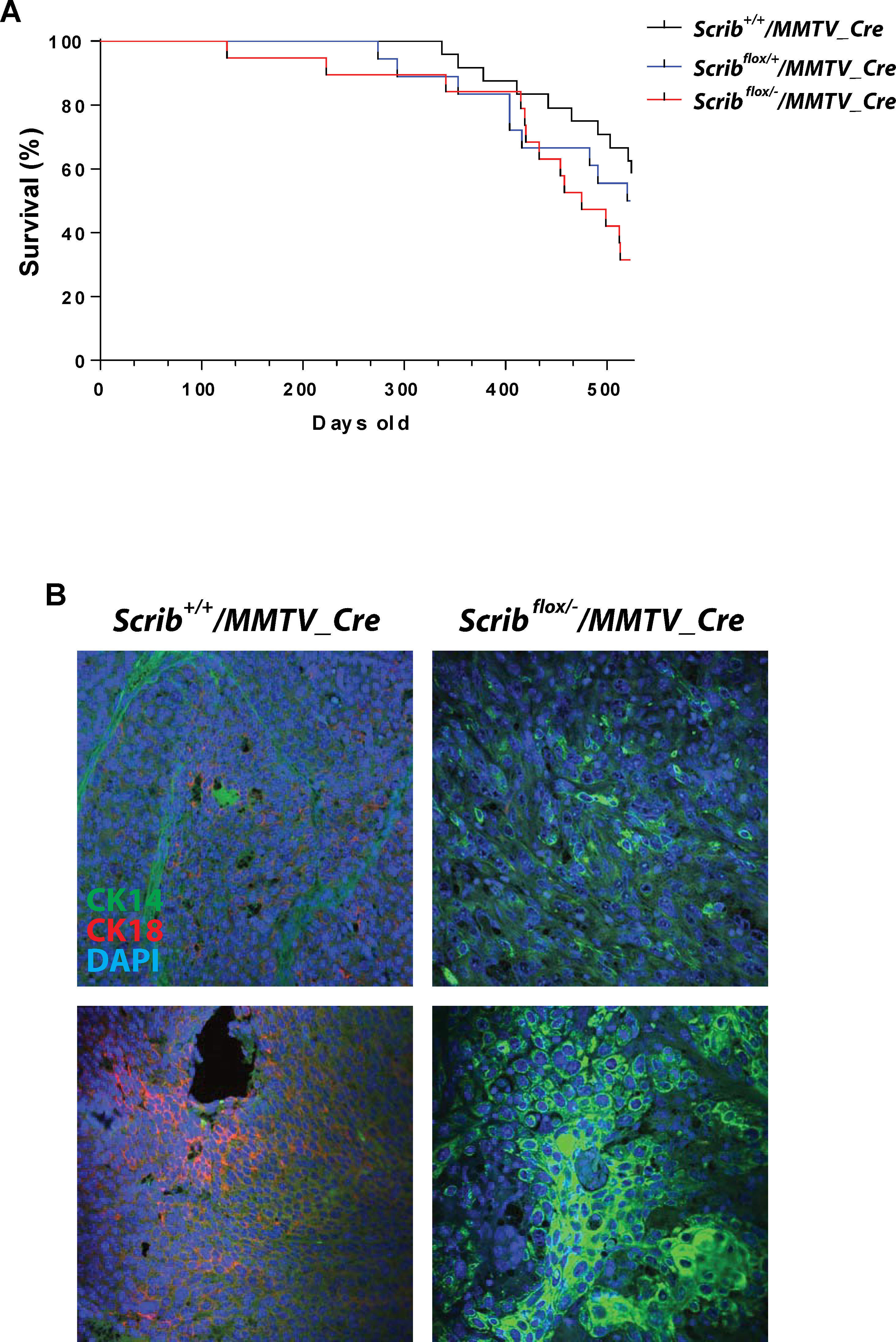

Supplement: Figure S6 — Survival analysis and tumour immunostaining in aged mice. A. Kaplan-Meir survival analysis for aged cohorts of MMTV-Cre (n = 24) versus MMTV-Cre;Scribflox/+ (n = 18) and MMTV-Cre;Scribflox/− (n = 19) virgin mice. Mice predominantly develop mammary tumors but also succumb to lung and ovarian tumors. B. Representative images of immunostaining of basal marker CK14 and luminal marker CK18 in tumors from MMTV-Cre and MMTV-Cre;Scribflox/− mice. (TIF) [file pgen.1004323.s006.tif]
