# Supplementary material for: Scribble Modulates the MAPK/Fra1 Pathway to Disrupt Luminal and Ductal Integrity and Suppress Tumour Formation in the Mammary Gland
Source: PLoS Genet. 2014 May 22;10(5):e1004323. doi: 10.1371/journal.pgen.1004323 (PMC4031063; doi:10.1371/journal.pgen.1004323)
Supplement: Procedures S1 — Experimental procedures for developmental staging, ultrastructural analysis, gene expression analysis and immunostaining. (DOCX) [file pgen.1004323.s009.docx]

**SUPPLEMENTAL EXPERIMENTAL PROCEDURES**

**Developmental stages**

The developmental stages examined were 6 week virgin, 12 week virgin, early pregnant (14.5 days post coital), late pregnant (16.5 days post coital), lactating (4 days post-partum), involution (4 days post weaning), 12 weeks post-pregnancy, and aged virgin (up to 525 days old). We found MMTV-cre mice on an FVB/n background exhibited stochastic mammary tumour formation with sufficient ageing. Expression of Cre transgenes promote genomic damage and thus sensitize mice to tumorigenesis ([Huh et al., 2010](#_ENREF_34); [Loonstra et al., 2001](#_ENREF_52)). Animals were taken at various dates and cages to minimize bias due to the estrus cycle. For pregnancy dependent studies animals were matured to at least 10 weeks age prior to mating and allowed only a single round of pregnancy.

**Ultrastructural analysis of mammary ducts**

Samples were prepared as described elsewhere ([Spurr, 1969](#_ENREF_84)). Briefly, tissue was fixed in 2% paraformaldehyde, 2.5% glutaraldehyde in 0.08 M Sorensen’s phosphate buffer (PBS) for 2 hours. Then washed in 3 X 10min changes of (PBS). Post-fixation was with 2% osmium tetroxide in PBS followed by dehydration through a graded series of alcohols, two acetone rinses and embedding in Spurrs resin (Spurr, 1969). Sections approximately 80nm thick were cut with a diamond knife (Diatome, Switzerland) on an Ultracut-S ultramicrotome (Leica, Mannheim, Germany) and contrasted with uranyl acetate and lead citrate. Images were captured with a Megaview II cooled CCD camera (Soft Imaging Solutions, Olympus, Australia) in a JEOL 1011 transmission electron microscope (TEM).

**Gene expression analysis**

Real time primers used were CK8F, 5’-GTCATACTGGGCAGGATGT-3’; CK8R, 5’-CAAGATCGAAGACCTGAGG-3’; αSMAF, 5’-CCCAGAGTGGAGAAGCCCAGC-3’; αSMAR, 5’-GCCCAGAGCCATTGTCGCAC-3’; Tcf4F, 5’- -3’; Tcf4R, 5’- -3’; CtnnbF, 5’-GAAACGGCTTTCAGTTGAGC-3’; CtnnbR, 5’-CTGGCCATATCCACCAGAGT-3’; Hey1F, 5’-TGAGCTGAGAAGGCTGGTAC-3’; Hey1R, 5’-ACCCCAAACTCCGATAGTCC-3’; Hey2F, 5’-TGCTCCAGGCTACAGGGGGTAA-3’; Hey2R, 5’-TCTGTGGCAAGAGCATGGGCA-3’; Hes6F, 5’-TGAGGACCGCTGGGAAGCAC-3’; Hes6R, 5’-TCTCGTTGATCCGTGCGCGT-3’; KitF, 5’-ATTGTGCTGGATGGATGGAT-3’; KitR, 5’-ATCTGCTCTGCGTCCTGTT-3’; Elf5F, 5’-GTGGCATCAAGAGTCAAGACTGTC-3’; Elf5R, 5’-CTCAGCTTCTCGTACGTCATCCTG-3’; GATA3F, 5’-ACGGTCAGCACCCAGACACG-3’; GATA3R, 5’-CCAGCCAGGGCAGAGATCCG-3’; cJunF, 5’-GGGACACAGCTTTCACCCTA-3’; cJunR, 5’-GAAAAGTAGCCCCCAACCTC-3’; Fra1F, 5’-CTCTTCCTCCTCTGGGCTG-3’; Fra1R, 5’-ATCCCCAGTACAGTCCCCCT-3’; GapdhF, 5’- TCCCACTCTTCCACCTTCGA-3’; GapdhR, 5’- GTCCACCACCCTGTTGCTGTA-3’.

**Immunofluorescence and Immunohistochemistry**

Additional antibodies used for Supplemental data include pAktS473 (4060, Cell Signaling Technology), pS6 (2211, Cell Signaling Technology), Npt2b (NPT2B11-A, Alpha Diagnostics), pPRAS40 (2997, Cell Signaling Technology). TUNEL kit (2156792, Roche Diagnostics) was used to quantify apoptosis during involution.
